# Estimation of Kinetics Using IMUs to Monitor and Aid in Clinical Decision-Making during ACL Rehabilitation: A Systematic Review

**DOI:** 10.3390/s24072163

**Published:** 2024-03-28

**Authors:** Sanchana Krishnakumar, Bert-Jan F. van Beijnum, Chris T. M. Baten, Peter H. Veltink, Jaap H. Buurke

**Affiliations:** 1 Department of Biomedical Signals and System, University of Twente, Drienerlolaan 5, 7522 NB Enschede, The Netherlands; b.j.f.vanbeijnum@utwente.nl (B.-J.F.v.B.); p.h.veltink@utwente.nl (P.H.V.); j.h.buurke@utwente.nl (J.H.B.); 2 Roessingh Research and Development, Roessinghsbleekweg 33B, 7522 AH Enschede, The Netherlands; c.baten@rrd.nl

**Keywords:** anterior cruciate ligament, ground reaction force, inertial measurement unit, joint kinetics, rehabilitation

## Abstract

After an ACL injury, rehabilitation consists of multiple phases, and progress between these phases is guided by subjective visual assessments of activities such as running, hopping, jump landing, etc. Estimation of objective kinetic measures like knee joint moments and GRF during assessment can help physiotherapists gain insights on knee loading and tailor rehabilitation protocols. Conventional methods deployed to estimate kinetics require complex, expensive systems and are limited to laboratory settings. Alternatively, multiple algorithms have been proposed in the literature to estimate kinetics from kinematics measured using only IMUs. However, the knowledge about their accuracy and generalizability for patient populations is still limited. Therefore, this article aims to identify the available algorithms for the estimation of kinetic parameters using kinematics measured only from IMUs and to evaluate their applicability in ACL rehabilitation through a comprehensive systematic review. The papers identified through the search were categorized based on the modelling techniques and kinetic parameters of interest, and subsequently compared based on the accuracies achieved and applicability for ACL patients during rehabilitation. IMUs have exhibited potential in estimating kinetic parameters with good accuracy, particularly for sagittal movements in healthy cohorts. However, several shortcomings were identified and future directions for improvement have been proposed, including extension of proposed algorithms to accommodate multiplanar movements and validation of the proposed techniques in diverse patient populations and in particular the ACL population.

## 1. Introduction

The anterior cruciate ligament (ACL) is one of the major stabilizing ligaments of the knee that ensures knee stability [1]. An ACL injury occurs when this ligament is stretched or torn and accounts for 64% of athletic knee injuries [1,2]. It commonly occurs in highly demanding sports such as soccer, volleyball, skiing, basketball, and football as they involve cutting, pivoting, and jumping actions [2]. Based on the complexity of the injury, patients are recommended either for conservative treatment with rehabilitation or reconstruction surgery. Regardless of the treatment decision, all these patients undergo rehabilitation. Rehabilitation after an ACL injury takes 9–12 months and usually consists of multiple phases based on the severity of the injury and end objective [3]. These phases include various dynamic exercises and tasks with different focuses like muscle strengthening, balance improvement, return to sports training and training for prevention of re-injury [3,4,5]. In everyday practice, physiotherapists assess the functional status of the patient through visual observations to devise a rehabilitation plan and to make phase transition decisions. Since the treatment is currently based on subjective visual observations during clinical visits, there is huge potential to further optimize the training of patients using quantitative assessment of relevant biomechanical parameters.

An initial literature review showed that knee moments and patellofemoral joint forces are important biomechanical parameters to be assessed during the ACL rehabilitation process [6]. In addition to measuring knee moments, ankle and hip moments may also be useful in designing training programs for athletes to prevent ACL injuries/reinjuries and to facilitate the return to sports programs [7]. However, physiotherapists cannot directly measure these parameters. This makes it important to use additional measurement tools during assessment by physiotherapists.

A traditional approach to computing joint kinetics is using a clinical gait lab with optical marker systems and force plates, which is also considered a gold standard system. Net joint moments can be computed with the traditional bottom-up inverse dynamics method using measured GRF and ground reaction moments (GRM). GRF and GRM can be measured using force plates and instrumented treadmills [8,9,10,11,12]. However, these systems have several disadvantages, as they are expensive and require extensive time for patient preparation, have bulky setups, and involve extensive manual post processing. Systems such as instrumented treadmills that measure GRF may also alter the natural pattern of gait [13,14]. On the other hand, force plates restrict measurements inside the lab to a few steps, making them not ideal for sports monitoring [15]. Another alternative to measuring ground reaction forces outside the lab setting are instrumented insoles. These insoles measure foot pressure and thereby estimate GRF [16,17]. However, they face technical challenges such as difficulty in accounting for frictional forces [18]. These insoles also have not been evaluated for their durability, especially for sports-like conditions, and measure only 1D GRF, while 3D GRF is important for monitoring sideward movements such as during ACL rehabilitation. Since ACL injuries predominantly occur in athletes, it is essential to monitor exercise/sports movements and to allow for measurements in remote settings, thereby making conventional measurement systems not suitable.

Inertial measurement units (IMUs) offer an alternative for quantitative gait analysis over optical marker systems. They are increasingly used in research, as they are small, portable, and suitable for outdoor and sports monitoring [19,20]. Their validity for kinematic gait analysis have been well studied and compared to optical reference systems [21]. Several algorithms also exist in the literature to estimate GRF and GRM, net joint moments, and other kinetic parameters using the measured kinematics from IMUs. However, their reliability is not well compared or studied. The algorithms in the literature can be grouped into three categories.

1. Algorithms that estimate GRF and GRM;
2. Algorithms that use a two-step approach, where GRF and GRM is estimated first and then the predicted GRF and GRM are used to estimate other kinetic parameters like net joint moments;
3. Algorithms that apply new approaches to directly estimate net joint moments and/or net joint forces.

Ancillao et al. [22] conducted a systematic review on the estimation of GRFs and GRMs using IMUs. The primary objective of that review was to identify and discuss methods to estimate GRF and GRM from IMU data. Algorithms published till the year 2018 in the first two categories were included in this review. Mundt et al. [23] in 2021 partly investigated the third category and compared three neural network (NN) approaches to estimate joint moments from IMUs: multilayer perceptron (MLP) networks, convolutional neural networks (CNNs), and recurrent neural network such as long short-term memory network (LSTM) for level-walking data. 

Currently, to the best of the author’s knowledge there are no reviews that identified algorithms in all three categories and evaluated them for their applicability in clinical decision-making for ACL rehabilitation. Thus, a systematic review that compares and evaluates available algorithms for estimation of kinetic parameters (joint kinetics, GRF, and GRM) using only IMU data will provide insights on the state of the art of the accuracy, reliability, and applicability of the available algorithms. In addition, it will help to identify the gaps and opportunities for further research and open new avenues for clinical decision-making for ACL rehabilitation and for other conditions. This might also be beneficial for other sports-related rehabilitation and injury prevention training. Therefore, the objective of this systematic review is to identify and discuss algorithms for the estimation of lower limb kinetic parameters only using IMUs without additional force information. To achieve this objective, the following research questions were formulated.

1. What are the available algorithms to estimate GRF and GRM using only IMU data as input and then use the predicted GRF and GRM to estimate lower limb joint kinetics?
2. What are the available algorithms to directly estimate lower limb joint kinetics using only IMU data as input?
3. What is the accuracy, reliability, and applicability of the identified algorithms for pathological gait and ACL-related tasks?

## 2. Materials and Methods

### 2.1. Study Design

This systematic review was conducted in accordance with the Preferred Reporting Items for Systematic Reviews and Meta-Analyses (PRISMA) statement (Supplementary Table S1) [24]. The steps included the formation of inclusion–exclusion criteria, pre-planned data analysis and extraction pipeline, and pre-defined quality assessment metrics. The methodology was registered in advance at the international database of systematic reviews PROSPERO (CRD42022304911) [25].

### 2.2. Search Strategy

The databases used for this review were PubMed, Scopus (Elsevier), and SPORTDiscus (EBSCO host). Three keyword groups were identified, and commonly accepted variations of the keywords were synthesized. A search query for each database was formulated using Boolean operator “AND” between the groups and “OR’ between the terms within the same group. Multiple successive searches were performed based on an expanded search query from previous search results. The finalized full search query is available in Appendix A (Table A1). The identified keyword groups were Group1: “lower extremity” AND ‘kinetics OR “ACL”, Group 2: “inertial measurement unit”, and Group 3: “algorithms”.

### 2.3. Inclusion/Exclusion Criteria

All original studies that used kinematic data from IMUs or simulated inertial data were included. Reviews, comparison studies that did not propose any new method or algorithms, abstracts, and veterinary studies were excluded. Studies were also excluded when they utilized force platforms, insoles, or instrumented shoes as external force input or electromyography (EMG) data as input for algorithms to estimate kinetic parameters. Studies were included only when the estimated kinetic parameters were compared to an existing reference system with known performance characteristics.

### 2.4. Study Selection and Quality Assessment

**Figure 1:**
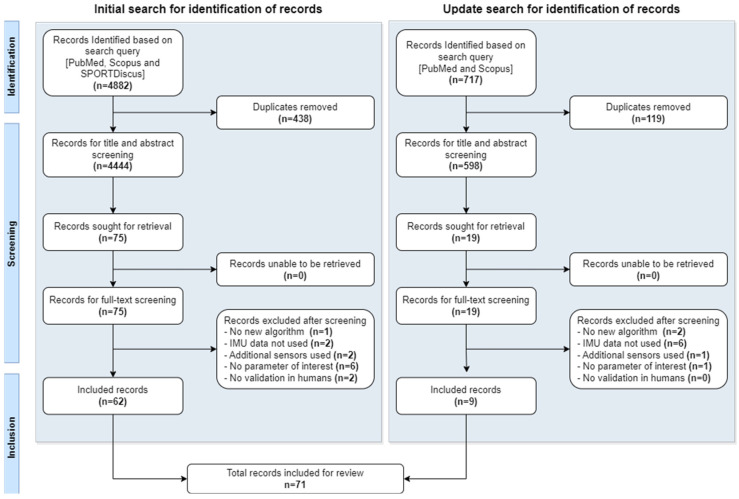
Systematic review process pipeline according to PRISMA.

Duplicate articles were identified and removed using reference manager EndNote 20.2.1 and the Rayyan web application (Rayyan System Inc., Cambridge, MA, USA), resulting in 4444 articles. The identified articles were then loaded into ASReview LAB 0.19 for screening (S.K). ASReview is an artificial intelligence tool that helps with identifying relevant papers. It requires a set of papers that will be included in the final review as a prior knowledge set and as a validation set. The tool then trains an active learning model based on the input data and produces papers based on order of relevance for the reviewer to perform title and abstract screening. The tool also retrains the model based on every subsequent user decision [26]. The screening was continued until 150 consecutive papers were marked irrelevant (chosen stop criteria). The title and abstract screening in ASReview (S.K) involved a total of 1082 articles and resulted in 187 relevant papers. To mitigate potential bias introduced by the first reviewer’s selections, 20% of the articles from the initial 893 excluded papers (1082–187) were added to Rayyan along with 187 papers for a consecutive title and abstract screening. Double-blinded abstract screening (S.K and B.-J.F.v.B.) of the identified articles was then performed using Rayyan. The inter-rater conflict was 19%, and the final decision of inclusion/exclusion was based on discussion between the reviewers until 100% agreement was reached. After abstract screening, 75 papers were included for full text screening. All articles obtained from the update search were manually screened (S.K), and 9 articles were included. Full-text screening was performed by the first reviewer (S.K), and 71 articles were included for quantitative data synthesis. The process pipeline for article screening and selection is provided in Figure 1.

Quality assessment of the included studies was performed (S.K) using a 14-point checklist comprising items listed in Table 1. Items 1, 2, 4, 6, 7, 8, 10, 11, 12, and 14 were directly replicated from Strom et al. [27]. Items 3 and 13 were modified and adapted from the same table to better align with the specific focus of this systematic review. Additionally, we added items 5 and 9 to address additional aspects relevant to our quality assessment. For detailed assessments and scores of all included studies, including explanations and descriptions of each item, please refer to Supplementary Table S2. 

### 2.5. Data Extraction

The data extraction was performed by first reviewer (S.K) using ATLAS.ti 23 for Windows. The following information was extracted to answer the research questions and sub-questions. General information on publication type, year, and author information was collected to gain an overview of all the papers. Information on study settings, methods, sensors used, population evaluated (healthy/pathological), sample size, and types of movements studied were extracted to understand the input requirements for the algorithms, identify gaps, and discuss the applicability of the algorithms. The type of algorithms proposed, including other information about the algorithms such as assumptions and processing steps, were also extracted. Estimated outcome parameters and their accuracy metrics in comparison to the reference system used were extracted. To ensure precision and accuracy in our review, we refrained from extracting information directly from graphical representations such as error plots. Instead, we focused on obtaining numerical values when explicitly provided in the text or abstract of the source papers.

## 3. Results

### 3.1. Publication Year

**Figure 2:**
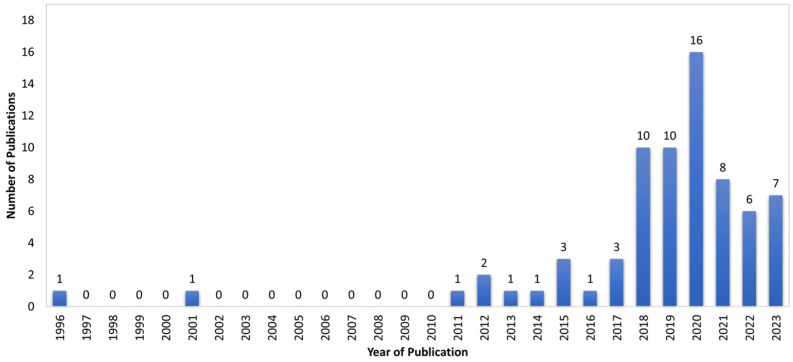
Number of publications per year.

The included articles were grouped based on year of publication and are depicted in Figure 2. The first publication found was from the year 1996 [28]. The highest number of included articles were published in 2020, with 16 publications. The year 2023 in Figure 2 only included articles published till 15 April 2023.

### 3.2. Participant Characteristics

**Figure 3:**
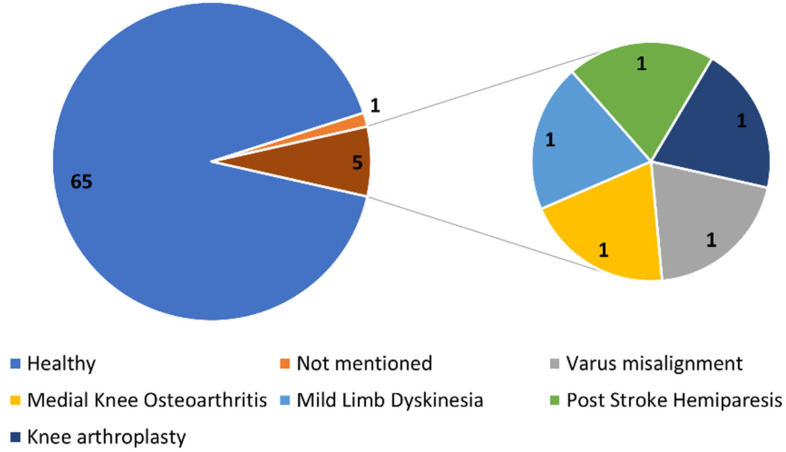
Participant demographics depicted along with the corresponding number of articles.

The validation of algorithms identified in the included papers was predominantly performed on healthy young adults and especially athletic cohorts (Figure 3). Only four articles (~6%) validated their algorithms on people with pathological gait [29,30,31,32]. Stetter et al. evaluated their algorithm on healthy volunteers with bowlegs, which was suggested to mimic the varus misalignment in patients with knee osteoarthritis [33]. Female population was underrepresented in 55 articles (~80%), while 6 articles (~8%) [30,32,34,35,36,37] did not report complete information on the gender distribution of the study population.

### 3.3. Measurement Systems and Sensor Placement

Among the included studies, most studies used tri-axial gyroscopes and/or triaxial accelerometers, and biaxial and uniaxial accelerometers were also used in few studies [36,38,39]. Information on sensor systems used, number of on-body sensors, and sensor characteristics are listed in Table 2. Not all studies reported the specifications of the sensor used; therefore, additional information was obtained through reference tracking and from the manufacturer website and is included in Table 2.

For validation of estimated kinetics, quantities like ground reaction forces were simultaneously measured in all studies using force plates [18,29,31,34,40,41,42,43,44,45,46,47,48], instrumented treadmills [14,32,38,39,49,50,51,52,53,54,55,56,57,58], plantar sensors [35], force shoes [59], shoes with loadcell(s) [60,61], or insoles [62,63,64,65]. Additional kinematic data were also measured in some of the studies using systems such as optical motion capture systems [30,33,36,37,66,67,68,69,70,71,72,73,74,75,76,77,78,79,80,81,82,83,84,85,86,87], a stereophotogrammetry system [88], or a marker-less video system [89], along with IMUs.

**Figure 4:**
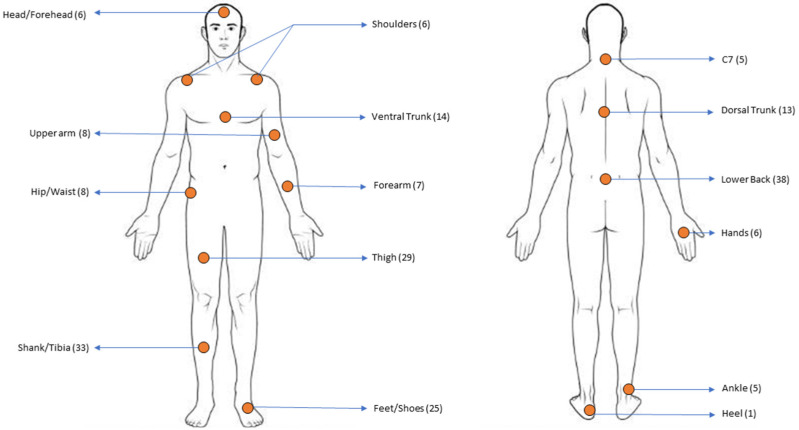
Sensor placement locations along with the corresponding number of articles. The ventral trunk placement location includes the sternum, chest, and torso. The dorsal trunk includes sensors placed on upper/mid-dorsal trunk. The feet included sensors placed on both the feet and the shoes. The lower back included sensors placed on the fifth lumbar vertebrae, pelvis, and sacrum.

The most common sensor placement location reported was the lower back, which was followed by the shank, thigh, and feet. The sensor locations used in the included studies are depicted in Figure 4. A few studies did not report the exact sensor placement locations; for these studies, the information on sensor locations was extracted from images. Most studies placed the sensors bilaterally on the lower limbs except for few studies that placed the sensors unilaterally (right/left lower limb) [32,33,36,38,47,48,51,53,57,66,68,83,85,86]. Studies that placed IMUs on a unilateral side either assumed bilateral symmetry [36] or chose the dominant/injured leg as the leg of interest [32]. A total of 28 articles [14,15,18,35,39,40,41,42,43,44,45,49,51,52,55,56,58,62,63,66,68,74,75,88,89,90,91] used fewer than 3 on-body sensors, where 19 of them [14,15,18,39,40,41,44,45,49,51,52,55,58,62,63,74,88,89,91] only used a single sensor to measure movement kinematics and to develop algorithms estimating kinetics. the second highest number of on-body sensors was 3, and they were found in 12 articles [29,34,36,38,47,48,57,59,64,65,85,92]. Full-body sensors, which consisted of 17 sensors, were seen in 6 articles [50,71,76,78,79,82]. Most of the studies (38 articles) measured movement with a sampling frequency in the range of 100–200 Hz [14,15,18,23,29,31,32,35,36,40,41,44,46,48,52,53,54,56,57,59,64,66,69,70,71,72,74,82,85,86,87,88,89,90,91,92,93]. The lowest sampling rate was found to be 40 Hz [39]. The highest sampling frequency of 2000 Hz was used by Alcantra et al. [38]; however, these data were then down sampled to 500 Hz for algorithm development. Further detailed information on sampling rates can be seen in Table 2.

**Table 2:** Sensor characteristics.

Ref. No.	Year	Sensor Type	Axis	No. of Sensors	Sampling Rate (Hz)	Other Specifications/Notes
[28]	1996	Accelerometer: EGAXT -*-10 (Entran Devices Inc., Fairfield, CT, USA)	Triaxial	4	300	N/A
[36]	2001	Accelerometer: ADXL05 (Analog Devices, Wilmington, DE, USA) and gyroscope: ENC 03J (Murata, Smyrna, GA, USA)	Uniaxial	3	100	±5 g, gyroscope response up to 50 Hz and range ±300 deg/s
[88]	2011	IMU: MTx (Xsens Technologies BV, Enschede, The Netherlands)	Triaxial	1	100	N/A
[39]	2012	Accelerometer: Biotrainer AM (IM Systems, Baltimore, MD, USA)	Bi axial	1	40	N/A
[91]	2012	IMU: MTx (Xsens Technologies BV, Enschede, The Netherlands)	Triaxial	1	100	N/A
[89]	2013	Accelerometer: SPI Pro (GPSports Pty. Ltd., Canberra, Australia)	Triaxial	1	100	±8 g
[61]	2014	IMU: MPU-6050 (InvenSense Inc., San Jose, CA, USA)	Triaxial	6	N/A	N/A
[60]	2015	IMU: MPU-6050 (InvenSense Inc., San Jose, CA, USA)	Triaxial	7	N/A	±2 g, ±250 deg/s and 0.01 deg
[37]	2015	IMU: customized sensor	Triaxial	8 + 2 (ski equipment)	400	±8 g accelerometer, ±1000/s gyroscope
[43]	2015	Accelerometer: MMA7260Q (Freescale Semiconductor Inc., Austin, TX, USA)	Triaxial	1 or 2	1000	±6 g
[87]	2016	IMU:BIOGEAR A60BP (Mizuno Corporation, Tokyo, Japan)	N/A	7	100	N/A
[79]	2017	IMU: MVN Link (Xsens Technologies BV, Enschede, The Netherlands)	Triaxial	17	240	N/A
[45]	2017	IMU: Yost (YEI Technology, Portsmouth, NH, USA)	Triaxial	1	450	±24 g, ±2000 deg/s
[92]	2017	IMU: Opal (APDM Wearable Technologies Inc., Portland, OR, USA)	Triaxial	3	128	±6 g
[40]	2018	Accelerometer: ActiGraph GT3X+ (ActiGraph LLC, Pensacola, FL, USA)	Triaxial	1	100	N/A
[41]	2018	Accelerometer: KXP94 (Kionex Inc., Ithaca, NY, USA) and GPS: Minimax XS4 (Catapult Innovation, Scoreby, Australia)	Triaxial	1	100	±13 g
[29]	2018	IMU: customized sensor	N/A	3	100	N/A
[50]	2018	IMU: MVN Link (Xsens Technologies BV, Enschede, The Netherlands)	Triaxial	17	240 (120)	N/A
[67]	2018	Simulated Inertial data				
[69]	2018	IMU: Opal (APDM Wearable Technologies Inc., Portland, OR, USA)	Triaxial	12	128 (100)	±16 g accelerometer, resolution 14-bit
[70]	2018	IMU: Opal (APDM Wearable Technologies Inc., Portland, OR, USA)	Triaxial	6	128 (100)	±16 g accelerometer, resolution 14-bit, dynamic accuracy 2.8°
[51]	2018	IMU: Opal (APDM Wearable Technologies Inc., Portland, OR, USA)	Triaxial	1	N/A	±6 g m/s^2^, ±2000 deg/s, and ±6 Gauss
[42]	2018	Accelerometer: MMA7260Q (Freescale Semiconductor Inc., Austin, TX, USA)	Triaxial	1 or 2	1000	±6 g
[54]	2018	IMU: Opal (APDM Wearable Technologies Inc., Portland, OR, USA)	Triaxial	6	128 (100)	N/A
[35]	2019	IMU: TSND151 (ATR-Promotions Co., Seika, Japan)	Triaxial	2	100	N/A
[30]	2019	Simulated Inertial data				
[68]	2019	IMU: customized sensor	Triaxial	2	1500	±8 g accelerometer, ±2000/s gyroscope
[90]	2019	Accelerometer: ActiGraph GT3X+ (ActiGraph LLC, Pensacola, FL, USA)	Triaxial	2	100	
[74]	2019	IMU: EBIMU-9DOFV4 (E2Box, Hanam, Gyeonggi-do, Republic of Korea)	N/A	1	100	±16 g and resolution of 0.001 g
[76]	2019	IMU: MTw Awinda (Xsens Technologies BV, Enschede, The Netherlands)	Triaxial	17	60	N/A
[78]	2019	IMU: MVN Link (Xsens Technologies BV, Enschede, The Netherlands)	Triaxial	17	240	N/A
[81]	2019	IMU: custom IMU ((Portabiles GmbH, Erlangen, Germany)	Triaxial	7	1000	±16 g accelerometer, ±2000/s gyroscope
[63]	2019	IMU+GPS: VN-200 (VectorNav Technologies, Dallas, TX, USA)	Triaxial	1	400	Velocity accuracy = ±0.05 m/s; inertial heading accuracy = 0.3° RMS; pitch/roll = 0.1° RMS; angular resolution < 0.05°; repeatability < 0.1°
[46]	2019	IMU: STT-IWS iSen (STT Systems, Gipuzkoa, Spain)	N/A	8	100	N/A
[59]	2020	IMU: MTw Awinda (Xsens Technologies BV, Enschede, The Netherlands)	Triaxial	3	100	N/A
[18]	2020	Accelerometer+GPS: MinimaxX S5 (Catapult Innovations, Scoresby, Australia)	Triaxial	1	100	±16 g, resolution 16-bit
[14]	2020	Accelerometer: GT9X Link (ActiGraph LLC, Pensacola, FL, USA)	Triaxial	1 or 2	100	12-bit ADC resolution, ±16 g range
[33]	2020	IMU: customized sensor	Triaxial	2	1500	±8 g accelerometer, ±2000/s gyroscope
[31]	2020	IMU: MTw Awinda (Xsens Technologies BV, Enschede, The Netherlands)	Triaxial	5 (3 + 2 for indirect)	100	N/A
[71]	2020	IMU: Perception Neuron Pro (Perception Neuron, Miami, FL, USA)	Triaxial	17	120 (100)	N/A
[72]	2020	IMU: MTw Awinda (Xsens Technologies BV, Enschede, The Netherlands)	Triaxial	4	100	±16 g accelerometer, ±2000 deg/s gyroscope
[73]	2020	Simulated inertial data but for validation used data from TinyCircuits, Akron, OH, USA, at 100 Hz				
[15]	2020	IMU: MTw Awinda (Xsens Technologies BV, Enschede, The Netherlands)	Triaxial	1	100	N/A
[44]	2020	IMU: Trigno Avanti sensors (Delsys, Natick, MA, USA)	Triaxial	1	148 (100)	N/A
[52]	2020	IMU: Opal v2 (APDM Wearable Technologies Inc., Portland, OR, USA)	Triaxial	1	128	±16 g
[77]	2020	Sacrum marker used to obtain velocity and acceleration of center of mass				
[53]	2020	IMU: MTw Awinda (Xsens Technologies BV, Enschede, The Netherlands)	Triaxial	4	100	N/A
[93]	2020	IMU: ActiGraph Link ((ActiGraph LLC, Pensacola, FL, USA)	Triaxial	6	100	N/A
[94]	2020	IMU + atmospheric pressure sensor:TSND151 (ATR-Promotions Co., Ltd., Kyoto, Japan)	Triaxial	7	N/A	N/A
[83]	2020	IMU: custom sensor (Portabiles GmbH, Erlangen, Germany)	Triaxial	4	1000	±16 g accelerometer, ±2000/s gyroscope
[23]	2021	IMU: TinyCircuits (TinyCircuits, Akron, OH, USA)	Triaxial	5	100	N/A
[62]	2021	IMU+GPS: VN-200 (VectorNav Technologies, Dallas, TX, USA)	Triaxial	1	400	Velocity accuracy = ±0.05 m/s Inertial heading accuracy = 0.3° RMS Pitch/roll = 0.1° RMS Angular resolution < 0.05° Repeatability < 0.1°
[75]	2021	IMU: MPU-6050 (InvenSense Inc., San Jose, CA, USA)	Triaxial	2	N/A	±8 g and 3-D gyroscope (±1000°/s)
[80]	2021	Simulated data from an old dataset. For the test set, data from IMU Noraxon DTS-3D 518 were used.				
[82]	2021	IMU: Perception Neuron Pro (Perception Neuron, Miami, FL, USA)	Triaxial	17	120	±16 g accelerometer, ±2000/s gyroscope
[84]	2021	IMU: Blue Trident (Vicon Motion Systems Ltd., Oxford, UK)	Triaxial	4	1125	Dual-g accelerometers (high: ±200 g, low: ±16 g), gyroscope (±2000°/s), and magnetometer (±4900 µT).
[85]	2021	IMU+Pressure sensor: Physiolog (Gait UP SA, Lausanne, Switzerland)	Triaxial	3	128	N/A
[55]	2021	Accelerometer: IMeasureU (IMeasureU, Centennial, CO, USA)	Triaxial	1	500	±16 g
[38]	2022	Accelerometer: customized sensor	Biaxial	3	2000 (500)	N/A
[49]	2022	IMU: Movesense (Suunto Oy, Vantaa, Finland)	Triaxial	1	208	9.4 g
[66]	2022	IMU: Yost (YEI Technology, Portsmouth, NH, USA)	Triaxial	2	200	Data were discarded and simulated from optical data
[47]	2022	Accelerometer: TSD109F (Biopac Systems Inc., Goleta, USA)	Triaxial	3	1000	N/A
[56]	2022	Accelerometer: Mini Wave plus (ZeroWire, Cometa, Italy)	Triaxial	2	143	±16 g accelerometer
[57]	2022	Accelerometer: GT9X Link (ActiGraph LLC, Pensacola, FL, USA)	Triaxial	3	100	±16 g range; sensor has primary and secondary accelerometer. Study used secondary accelerometer that provides unfiltered output.
[86]	2023	IMU: Yost (YEI Technology, Portsmouth, NH, USA) and SageMotion (Sagemotion Inc., Waterside, NB, Canada)	Triaxial	Dataset 1: 4 Dataset 2: 8	Dataset 1: 200 Dataset 2: 100	Dataset 1: N/A Dataset 2: resolution 0.1 m/s^2^, 0.06 deg/s
[64]	2023	IMU: Casio (Casio Computer Co., Ltd., Tokyo, Japan)	Triaxial	3	200	0–16 g
[32]	2023	IMU: Physiolog 4 and 5 (Gait UP SA, Lausanne, Switzerland)	Triaxial	5	200	N/A
[48]	2023	Accelerometer: GT9X Link (ActiGraph LLC, Pensacola, FL, USA)	Triaxial	3	100	±16 g range; sensor has primary and secondary accelerometer. Study used secondary accelerometer that provides unfiltered output.
[34]	2023	IMU: BioStampRC BRCS01 (MC10 Inc., Cambridge, MA, USA)	Triaxial	3	250	±16 g accelerometer, ±2000/s gyroscope
[58]	2023	IMU: Movesense (Suunto Oy, Vantaa, Finland)	Triaxial	1	208	±8 g
[65]	2023	IMU: Casio (Casio Computer Co., Ltd., Tokyo, Japan)	Triaxial	3	200	N/A

N/A—Not available.

### 3.4. Activities Studied

**Figure 5:**
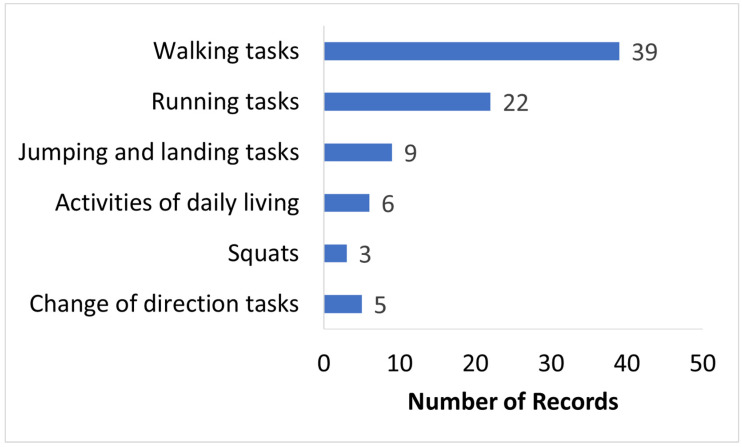
Activities measured in the included articles.

Activities such as walking, running, squats, jumping, and activities of daily living were measured and investigated in the included articles as seen in Figure 5. Most of the included articles studied walking and running activities. For walking, both overground walking [15,23,28,31,35,36,39,42,43,59,60,61,62,68,73,75,76,77,78,79,81,82,83,86,90,92,94] and treadmill walking [14,32,40,44,53,54,56,57,69,70] were studied in 29 and 10 articles, respectively. Among the walking studies, a few of them also included variations in walking [15,40,59,68,75,81,90] such as asymmetrical walking, walk and turn, and slalom walking. For running, both overground running [18,28,33,39,41,45,64,65,67,68,80,82,83,89] and treadmill running [38,49,50,51,52,55,56,58] were studied in 14 and 8 articles, respectively. Activities of daily living (ADL) included tasks such as sit to stand (STS) [29,87], stair ascent and decent [76,86], manual material handling [82], and standing balance [72]. Jumping activities studied in the articles included unilateral and bilateral jumps during ballet [93], ski jumps [37], skipping [71], vertical drop jumps [46,47,48,84,85], and continuous jumps [48]. Few of the included articles also measured sports-related activities relevant to ACL rehabilitation such as squats [87,88,91], side-to-side jumps [71], vertical drop jump and landing [46,47,48,84,85], countermovement jumps [34], and change-in-direction tasks [33,45,67,80,89] such as the cutting maneuver [33] and football alternate jumps [46]. Activities of similar movements were grouped and represented in the articles.

### 3.5. Types of Algorithms and Estimated Parameters of Interest

Based on the approach taken, several types of models used to estimate kinetics can be identified. The four main types we identified are (i) biomechanical models, (ii) musculoskeletal models, (iii) statistical models, and (iv) machine learning models.

Biomechanical (BM) models in this context refer to algorithms that use kinematics measured from IMUs along with inertial properties of (rigid) body segments and their biomechanical relationships to compute kinetics by applying Newton–Euler motion equations. However, these equations become indeterminate during double support when the distribution of load between the feet is unknown. Additional functions like the smooth transition approach were introduced to deal with this problem [79], but some articles only focused on estimating GRF during single support.

Musculoskeletal (MS)-based modelling approaches extend biomechanical-based models that only include rigid segment modelling by including properties of muscles, bones, and ligaments. The utilization of a combination of IMU measured kinematics and MS modelling enables the estimation of muscle forces along with joint kinetics. The parameters estimated using MS modelling-based methods included GRF and GRM [78], GRF [71,81], joint reaction forces [76,78], and joint moments [76,78,81].

Statistical modelling (SM) relies on finding relationships between measured kinematics from IMUs and measured kinetics from the force plate to build prediction equations using regression-based techniques. Knowledge of basic biomechanical relationships between measured kinetics and kinematics is used to select inputs and identify relationships between them. These methods were built to estimate mainly peak vertical GRF (pVGRF), peak GRF (pGRF), peak loading rate, GRF breaking, and propulsion point metrics from accelerometer data [14,31,39,40,48,57,90]. However, Chien et al. used backward regression analysis to estimate the full GRF profile [47].

**Figure 6:**
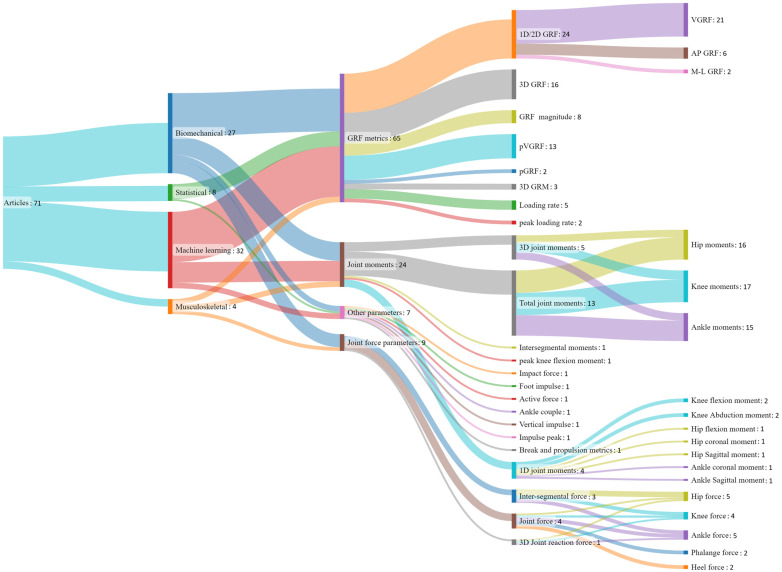
Sankey diagram representing several types of models used and all estimated kinetic parameters.

Machine learning (ML)-based methods are data-driven models to analyze the patterns and relationships between the measured kinematics from IMUs and target biomechanical variables calculated from laboratory-based systems without explicit programming, which is particularly useful for finding nonlinear relationships. Among the articles that utilized ML, various models were implemented and evaluated for estimating kinetics, including artificial neural network (ANN) [33,50,51,58,63,68,73,74,77,93], MLP [18,23,42,43], LSTM [23,30,64,66], bi-directional LSTM (BD-LSTM) [65], CNN [23,80,83], feed-forward neural network (FFNN) [30,44], fully connected neural network (FCNN) [66], temporal convolutional network (TCN) [66], recurrent neural network (RNN) [38], linear regression (LR) [55,58], stepwise linear regression (SLR) [84], random forest (RF) [53], quantile regression forest (QRF) [55], reservoir computer (RC) [32,56], support vector regression (SVR) [58], deep neural network (DNN) [35,62], nonlinear autoregressive neural network (NARX-NN) [85], nonlinear auto-regressive moving average model (NARMAX) [92], and deep learning (DL) [86]. An overview of all estimated kinetic parameters from the identified models is presented in Figure 6.

### 3.6. Accuracy and Reliability of Tested Approaches

All reviewed models were compared to a reference system of known characteristics using various metrics such as root mean square error (RMSE) [14,15,18,28,29,31,33,36,37,38,41,44,45,49,50,51,52,59,60,63,66,67,71,72,73,75,76,78,79,81,82,83,87,90,93,94], normalized root mean square error (NRMSE) [15,23,30,44,62,73,74,86,88,91,95], relative root mean square error (rRMSE) [33,38,49,79,82], Pearson correlation coefficient [15,29,42,43,50,58,76,79,81,83,86,93], Pearson product moment correlation [45], correlation coefficient [18,23,30,72,73,87,88,91], Spearman correlation coefficient [89], cross-correlation coefficient [51], interclass correlation coefficient (ICC) [82], mean absolute percentage error (MAPE) [14,38,48,57,58], max error [29,60], mean absolute error (MAE) [14,32,44,48,57,74,90], mean absolute deviation (MAD) [42,43], percentage difference [28,35,39,68], absolute percentage difference [39,40], and absolute difference [15,89]. The accuracy metrics used to evaluate the accuracy of the estimated parameters varied between kinetic parameters, although RMSE-based metrics were the most used accuracy evaluation metrics. RMSE-based metrics and correlation coefficients were the most used metrics to evaluate continuous parameters like GRF and joint moment estimates. Besides RMSE metrics, absolute difference and other difference related metrics were also commonly used for evaluating accuracies of discrete parameters, such as pGRF and pVGRF predictions. Estimated parameters were grouped into four categories: GRF and GRM, peak GRF metrics, joint moments, and other force metrics. Individual accuracy comparison tables were drafted for comparison, as seen in Supplementary Tables S3, S4, S5, and S6, respectively. For 3D GRF, the best RMSE values were achieved for vertical drop jump, namely, 0.018, 0.008, and 0.038 (normalized to body weight) for anterior–posterior GRF (APGRF), medio-lateral GRF (M-LGRF), and vertical GRF (VGRF) respectively [85]. The lowest rRMSE achieved for models that estimated both 3D GRF and 3D GRM was for the walking task. This was 9.40%, 13.10%, and 5.30% for APGRF, M-LGRF, and VGRF, respectively, and 29.60%, 12.40%, and 17.20% for frontal GRM (FGRM), sagittal GRM (SGRM), and transverse GRM (TGRM), respectively [79]. The most accurate estimates of pVGRF, with RMSE values ranging between 0.12 and 0.14 (normalized to body weight), were consistently observed during running activities [49,55,58]. On the other hand, the lowest RMSE for pGRF was 0.076 (normalized to body weight) and was observed during walking [90]. Among the articles that estimated 3D net knee joint moments, the lowest nRMSE (%) was observed for walking, with values of 10.58, 9.46, and 17.12 for abduction–adduction, flexion–extension, and external/internal rotation moments, respectively [30].

## 4. Discussion

The main objective and scope of this systematic review was to identify existing algorithms to estimate kinetic parameters using IMU data and to evaluate their accuracy, applicability, and reliability for ACL rehabilitation. A structured approach was taken to address the objectives, which started with a preliminary literature search to identify relevant kinetic parameters for ACL rehabilitation. Following this, a systematic literature search and data extraction and synthesis were conducted and reported. A significant increase in the number of papers was seen after 2017. We found that a significant number of new algorithms have been proposed since the last available systematic review related to estimation of ground reaction forces using IMUs in 2018, and therefore, this review provides several new insights for estimating GRFs and GRMs and adds new information on existing algorithms for estimating joint kinetics.

### 4.1. Modelling Techniques and Estimated Kinetic Parameters

The majority of the reviewed articles utilized ML-based models and accounted for around 45% of the reviewed articles, followed by BM (~38%). All four types of proposed algorithms have their own advantages and disadvantages. The SM approach provides methods to estimate kinetic parameters and can also help with studying various interaction effects on the estimation of kinetic parameters. Authors who utilized SM often also studied the effects of age, body mass, and sex on the prediction of kinetics [39,40,48,90]. The use of SM approaches was primarily observed for the estimation of 1D GRF and peak GRF metrics. Additionally, some studies utilized SM approaches to estimate kinetic parameters derived from GRF, such as loading rate [14,48,57] and propulsion and break metrics [31]. Although these parameters offer several useful insights, they were not used to estimate 3D continuous kinetic parameters for various dynamic activities. BM and MS are based on well-established principles of human anatomy and therefore also provide meaningful interpretations of the obtained metrics. However, BM is often sensitive to the input data, such as measured anthropometric data and generalized anatomical properties, which can affect the prediction outcomes. BMs are also built based on multiple assumptions about specific gait patterns and the standard anthropometric characteristics of a healthy population, rendering questionable their adaptability to diverse types of movement that were not studied and for patient groups.

ML-based models, on the other hand, can handle complex relationships in data and can be trained on diverse datasets, making them more generalizable, but these often come at the cost of requiring large, high-quality datasets for training. This can be challenging to obtain, especially when concerning patient populations. Some strategies for creating new inertial data from existing datasets were adopted to increase the size of the datasets in some of the reviewed articles [30,73,77,80]. According to Verheul et al. [67], utilizing ML-based techniques must be carried out with caution, as they are computational-based methods and thus may not account for and explain underlying physiological mechanisms. Although BMs considers physiological mechanisms, they still do not account for muscle forces or activations. MS-based models, on the other hand, account for muscle properties and forces and therefore may model physiological mechanisms more accurately. This property of MS-based models made it possible to estimate joint reaction forces [76,78].

Intersegmental forces and forces on joints were estimated only by BM and MS models, except for [68], where Stetter et al. used ML modelling (ANN) to estimate knee joint forces. Three-dimensional GRM was only estimated in three studies [78,79,80] using BM, ML, and MS models. The limited number of studies in the literature for the estimation of GRM highlights its potential for future research in this area. It is particularly important because GRM is an important input for understanding how GRF generates moments at a particular joint. The importance of GRM along with GRF as an input for the estimation of joint kinetics was also discussed by Karatsidis et al. [79].

### 4.2. Modelling Techniques, Tasks Studied, and Accuracies of Estimated Kinetic Parameters

In general, the large variety of accuracy metrics and their differences, along with the various activities studied, makes it challenging to assess the accuracies and compare the performance of the different proposed models in the reviewed articles. Sharma et al. [62] also discussed that it is difficult to compare the accuracies of various approaches from the literature due to differences in experimental methodologies (activities tested, conditions, and equipment used). In general, the highest accuracies achieved for estimation of kinetics were based on ML models, followed by BM models. It is important to note that the same algorithm, when validated for a particular activity at varying speeds, resulted in different accuracies. Activities performed at a comfortable a pace resulted in the best performance of the reported models, while the errors noticeably increased for higher and lower speeds [36,41,44,49,51,53,67]. However, this trend was not found in [74,77], where fast walking resulted in a more accurate estimation than normal walking, although slow walking resulted in higher NRMSE and RMSE. This may be because of a decrease in dynamic range of activity during slow movements. To better understand the effect of varying speeds on accuracies, the use of IMUs should be studied first at the kinematic level for the effect of variations in speed and then compared to optical marker systems in future studies. 

All articles that achieved the lowest RMSE/rRMSE for the estimation of 3D GRF, 3D GRM, pGRF, and pVGRF studied either walking or running, except Cerfoglio et al., who studied vertical drop jump [85]. It is also important to note that all these activities take place primarily in the sagittal plane and have the highest magnitude of GRF in the vertical direction, while M-LGRF and APGRF are of lower magnitude. Additionally, in most of the papers, M-LGRF and APGRFs were indicated to have lower RMSEs compared to VGRFs. However, the magnitudes of M-LGRF and APGRF are significantly lower than VGRF, and therefore, it is important to look at metrics that provide a normalized view of the errors considering the variability of data such as rRMSE and nRMSE.

Side-to-side jump was studied by Recinos et al., but only VGRF was estimated, with an RMSE of 0.108–0.274 (normalized to bodyweight) [71]. Johnson et al. [80] proposed ML algorithms to estimate 3D GRF and 3D GRM for running and sidestepping activities. However, only correlation coefficients of the models were reported. “Good” correlation greater than 0.85 was found for 3D GRF and “moderate” correlation between 0.6 and 0.7 was found for 3D GRM. Therefore, it would be beneficial to evaluate the applicability of these models to estimate 3D GRF for multiplanar tasks. A similar observation can be made for the estimation of joint moments.

Chaaban et al. [84] validated their approach of predicting VGRF and knee kinetics in healthy volunteers. They compared their computed RMSE values against differences between reported kinetics values of ACL patients and healthy volunteers from the literature (also termed “clinical difference”). These clinical differences reported were 0.24 and 0.035 for VGRF and knee flexion–extension moment, respectively. They suggested that since their model achieved an RMSE of 0.21 and 0.027 for VGRF and knee flexion–extension moment, respectively (lower than the clinical difference), their algorithm is appropriate. However, asymmetries and other factors in patients may lead to lower accuracies when validated for ACL patients.

### 4.3. Effect of Sensor Location and Sensor Characteristics on the Accuracy of Estimated Parameters

Around 56% of the included articles (40 articles) used three or fewer than three on-body sensors. Only 6 articles used 17 sensors and measured whole-body kinematics. Apparently, using a minimal set of sensors was given priority. Although some articles used multiple sensors, they explicitly tried to find a minimal and optimal sensor set. Multiple walking studies reported the pelvis sensor to be the most accurate [32,93]. Although the pelvis is known to be a good sensor location for estimating GRF due to the assumption of being close to the body’s center of mass, Havashinezhadian et al. [32] discussed that this assumption may not be valid for aging populations and pathological gait such as in knee osteoarthritis due to changes in upper-body stability. Havashinezhadian et al. [32] reported that the top of the shoe is the best location to predict 3D GRF in medial knee osteoarthritis patients. The seventh cervical vertebra was also reported to be a good sensor location for the estimation of VGRF [70]. Revi et al. used three sensors (pelvis, thigh, and shank) to estimate APGRF and found that even the removal of one of the sensors significantly affected the model’s accuracy in both healthy and post-stroke patients. Kerns et al. studied countermovement jumps using a sensor on the trunk and found that trunk movement during countermovement jump caused an increase in error estimates [34]. Therefore, it is important to consider the parameters of interest, type of task to be measured, and patient population characteristics when deciding on an optimal sensor placement.

Along with the choice of best placement location, the repeatability of IMU placements is also critical for accurate and repeatable kinetics estimations, Tan et al. studied the influence of IMU misplacement errors on GRF prediction and found that position misplacement of one sensor caused only a 1% difference; however, when eight sensors were misplaced, it lead to an error in GRF estimation of 4–6%. Error in the change in orientations of eight IMUs caused a difference of 20% in estimated 3D GRF. It is therefore suggested by the author that IMU placements are critical to obtain accurate data from the accelerometer and gyroscope for kinetic estimations. However, these errors can be compensated for by applying additional steps like rotating the sensor data to a segment frame using a static, helical axis or any other “body segment” calibration [96,97].

It was observed that 27 of the included articles (38%) measured activities at a sampling rate of 100–200 Hz; however, Sharma et al. [62] suggested that this may not be good enough to track dynamics during highly dynamic activities like sprint running, where about five steps per second could be observed. They acquired kinematic data at 400 Hz and stated that it was sufficient for their measurements. This was also consistent with other included studies that had higher sampling rates that measured dynamic activities such as running, jumping, and landing tasks. However, three studies that studied walking also used a higher sampling rate (400–1000 Hz) [42,62,81]. Therefore, sampling rates higher than 100–200 Hz may be needed for application in ACL rehabilitation, as it involves dynamic activities such as change in direction, jumping, and landing tasks. However, the sampling frequency of 100 Hz would be sufficient for other movements such as walking, double-leg, and single-leg squats.

### 4.4. Applicability for ACL Rehabilitation

To assess the applicability of the proposed algorithms in the literature, it is crucial to not just consider the achieved accuracies but also consider the validated activity and its generalizability on patient populations. In Figure 3, we could see that most of the articles were only validated on healthy cohorts, except for four studies [29,30,31,32]. The accuracies of the reviewed approaches may be different in patients when compared to healthy cohorts. This is also supported by the results of Liu et al. [29], who studied STS movements in both healthy people and people with mild lower-limb dyskinesia. The VGRF estimates had higher RMSE and correlation coefficients in the patient group compared to healthy volunteers. Liu et al. discussed that the differences are because the patients performed the tasks at a slower pace that increased the muscle tremble and sway in body motion, which in turn contaminated the measured signal, causing lower accuracies. Some authors studied and validated their algorithms on asymmetrical gait movements and found lower accuracies compared to accuracies achieved on normal movement [15,59,75]. These results indicate that it is important to validate the algorithms on pathological gait, as the assumptions of the algorithms may not be valid for patient groups and therefore may result in lower accuracies. It is also known from the literature that ACL patients have increased knee flexion for at least a year after reconstruction [98,99], and hence, it is important to validate the models with data from ACL patients. However, none of the articles included in the study validated their algorithms on ACL patients.

Many of the included articles (54%) only studied walking. Important ACL rehabilitation-specific tasks such as single-leg hop and triple hop have not been studied. Change-in-direction tasks could be more extensively studied to include multiplanar movements. Another important finding is that the female population was underrepresented in 80% of the included articles. Neugebauer et al. studied the estimation of GRF in youth during walking and running and discussed that sex is an influencing factor in the estimation of GRFs [39]. It is also important to note that the female population has an increased risk of ACL injury [100]. The increased risk of ACL injury in female populations may be attributed to neuromuscular differences such as a reduced effectiveness in stiffening the knee, causing increased tibial translation [101]. Therefore, it is essential to have equal female representation in the validation process. Additionally, it may also be useful to come up with different strategies or methodologies to account for gender and other anthropometric differences to ensure the generalizability of the proposed algorithms. The sample size of participants studied also differed significantly among the included studies. The median sample size was 12 participants, with individual sample sizes ranging from 1 [28,35,71,94] to 131 [57] participants. A total of 25 articles (35%) [15,28,29,34,35,36,37,50,51,53,59,60,61,62,63,69,70,71,74,76,77,82,87,92,94] involved fewer than 10 participants. It is important to ensure that the considered sample size has enough statistical power to detect meaningful differences and obtain meaningful results. 

A power analysis was conducted using G*Power 3.1.9.7 for a *t*-test comparing two independent means with an alpha = 0.05, a power of 0.80, and a medium effect size of 0.5. The chosen values were based on the work of Shirazi et al. [102]. The computed required sample size was 51. Only 8 of the included papers in this study tested more than 51 participants [14,23,48,49,54,57,58,90]. It is important to recognize that the calculated sample size serves as an indicator rather than a definitive guideline, as actual sample sizes can vary based on several factors and are largely dependent on the type of study design and population. Therefore, future research should aim to establish sample sizes aligned with expected effect sizes and carefully evaluate the statistical significance of their findings. 

### 4.5. Limitations of the Included Evidence, Review Process, and Future Directions

Although this systematic review was conducted comprehensively and methodologically, it is also crucial to acknowledge the potential bias and constraints that may influence the generalizability of the results. Firstly, although the articles were diligently searched and queried in three of the major databases, there may be a few articles that were missed due to indexing, resulting in inclusion bias. The articles included in the review were also limited to only English. The decision to include only articles that validated on human beings while improving the quality of the included results may have limited the inclusiveness of the review.

The use of varying sensor placement locations, experimental protocols, and reporting metrics used in the included articles made direct comparison and identification of the overall best model with the most accurate outcome challenging. Also, some articles only validated a small number of subjects, bringing their reliability and generalizability into question. Therefore, it is important to consider these limitations when drawing conclusions about the implications of the results. To address these challenges, future research should adhere to standardized methodologies such as maintaining an adequate sample size, choosing the right validation groups, employing standard experimental protocols, and choosing appropriate accuracy metrics. Furthermore, this also emphasizes the need for large, diverse, open datasets involving both patients and healthy subjects performing activities relevant to ACL rehabilitation. This would enable efficient comparison of the testing of existing and new algorithms. Such efforts would be crucial for facilitating comparisons across studies in the literature as well as enhancing the relevance and applicability of research findings.

## 5. Conclusions

In this comprehensive systematic review, we aimed to assess the current state of the art in using IMUs to estimate GRF and joint kinetics, specifically focusing on their potential use in ACL rehabilitation. The results of this review indicate that IMUs have good potential to estimate GRF and other joint kinetic parameters with good accuracy for movements primarily in the sagittal plane for healthy cohorts. However, none of these algorithms have been validated on ACL patients. Kinetic estimations for multiplanar ACL-relevant movements such as side hops or change-in-direction tasks have not been well studied. Combining multiple models such as BM along with ML-based techniques could help overcome their individual limitations and therefore help achieve more accurate estimates of joint kinetics and GRF. Gaining a deeper understanding of kinetic parameters and their implications can help clinicians assess ACL patients during rehabilitation and can also be extended to a broader rehabilitation context for other conditions. Additionally, it can also help build tailored gait-retraining strategies and develop future injury prevention techniques during athletic training.

## Figures and Tables

**Table 1:** Quality assessment checklist.

Item	Description	Outcome
Aim of Work		
1	Description of a specific, clearly stated purpose (IV)	1, 0.5 or 0
2	The research question is scientifically relevant (EV)	1, 0.5 or 0
Inclusion Criteria (Selection Bias)		
3	Description of inclusion and/or exclusion criteria along with information about volunteers and/or patients (IV/EV)	1, 0.5 or 0
Methods (Performance Bias)		
4	Data collection is clearly described and reliable (IV/EV)	1, 0.5 or 0
5	Description about activities measured, validation tasks, warmup (IV/EV)	1, 0.5 or 0
6	Data processing is clearly described and reliable (IV/EV)	1, 0.5 or 0
7	Algorithms are clearly described and referenced (IV/EV)	1, 0.5 or 0
Outcomes (Detection Bias)		
8	Outcomes are topic relevant (EV)	1, 0.5 or 0
9	Types and variety of tasks validated with respect to ACL rehabilitation and sports monitoring	1, 0.5 or 0
10	The work answers the scientific question stated in the aim (IV)	1, 0.5 or 0
Presentation of the Results		
11	Presentation of the results is sufficient to assess the adequacy of the analysis (IV)	1, 0.5 or 0
12	The main findings are clearly described (IV)	1, 0.5 or 0
Data Analysis and Statistical Approach		
13	Appropriate statistical and comparison techniques to compare results with reference-to-reference system (SV)	1, 0.5 or 0
14	Sufficient number of subjects (SV)	1, 0.5 or 0

Note: IV—internal validity, EV—external validity, SV—statistical validity. Outcomes 1, 0.5, and 0 correspond to “complete”, “partially met”, and “not complete”, respectively. The above table was adapted based on the work of Strom et al. [27].

## Supplementary Materials

The following supporting information can be downloaded at: https://www.mdpi.com/article/10.3390/s24072163/s1. Table S1: PRISMA 2020 Checklist; Table S2: Quality assessment Checklist; Table S3–S6: Accuracy Tables for estimated kinetic metrics from the included papers.

## Abbreviations

ACL	Anterior cruciate ligament
ANN	Artificial neural network
APGRF	Anterior–posterior ground reaction force
BD-LSTM	Bi-directional long short-term memory network
BM	Biomechanical
CNN	Convolutional neural networks
DL	Deep learning
DNN	Deep neural network
EMG	Electromyography
FCNN	Fully connected neural network
FFNN	Feed-forward neural network
GRF	Ground reaction force
GRM	Ground reaction moments
ICC	Interclass correlation coefficient
IMU	Inertial measurement units
LR	Linear regression
LSTM	Long short-term memory network
MAD	Mean absolute deviation
MAE	Mean absolute error
MAPE	Mean absolute percentage error
M-LGRF	Medio-lateral ground reaction force
MLP	Multilayer perceptron
MS	Musculoskeletal
NARMAX	Nonlinear auto-regressive moving average model
NARX-NN	Nonlinear autoregressive neural network
NN	Neural network
NRMSE	Relative root mean square error
pGRF	Peak ground reaction force
pVGRF	Peak vertical ground reaction force
QRF	Quantile regression forest
RC	Reservoir computer
RF	Random forest
RMSE	Root mean square error
RNN	Recurrent neural network
rRMSE	Normalized root mean square error
SLR	Stepwise linear regression
SM	Statistical model
SVR	Support vector regression
TCN	Temporal convolutional network
VGRF	Vertical ground reaction force

## Appendix A

**Table A1:** Search query and filter settings for Scopus, PubMed, and SPORTDiscus.

**Scopus** [Title and abstract search] *Last Accessed: 15 April 2023*		(TITLE-ABS (((anterior) W/3 (cruciate) W/3 (ligament)) OR ((lower) W/3 (extremit* OR limb*)) OR ((ankle* OR foot OR feet OR leg OR legs OR knee OR knees OR thigh* OR lower-leg* OR joint OR joints) W/3 (kinetic* OR dynamic* OR moment* OR load* OR force* OR mechanic* OR reaction)) OR ((ground*) W/2 (reaction*) W/2 (force* OR moment* OR torque*)))) AND (TITLE-ABS(((wearable* OR body-worn*) W/3 (electronic* OR device* OR sensor* OR motion*)) OR ((wireless* OR inertial* OR IMU OR IMMU OR MIMU) W/3 (sensor* OR device* OR unit* OR data OR capture* OR analys*)) OR ((ambulat*) W/3 (monitor*)) OR ((3D OR 3-d OR 3-dimen* OR threedimension* OR three-dimension*) W/3 (sensor* OR motion* OR analys*)) OR accelerometer* OR accelero-meter*)) AND (TITLE-ABS(algorithm* OR algorythm* OR ((early) W/3 (ident* OR risk*)) OR AI OR automated-reason* OR computer-heurist* OR ((bayes*) W/3 (combiner* OR network*)) OR ((nearest) W/3 (neighbo*)) OR ((random*) W/3 (forest*)) OR ((support*) W/6 (vector*) W/6 (machine* OR active)) OR ((extract*) W/6 (feature*) W/6 (vector*)) OR ((artificial* OR ambient* OR machine* OR algorithm* OR neural) W/3 (intelligen* OR network*)) OR ((automat*) W/3 (pattern*) W/3 (recogni*)) OR ((learn*) W/3 (algori*)) OR ((machine* OR deep OR supervis* OR unsupervis* OR bayes) W/3 (learn*)) OR predict* OR estimat* OR comput* OR calculate* OR Anybody OR Opensim OR calibration* OR crossvalidat* OR validat* OR ((probab* OR predict*) W/3 (system* OR model* OR score* OR scoring*)))) AND DOCTYPE (ar) AND (LIMIT-TO (LANGUAGE, “English”))
**PubMed** [Filters—language: English] *Last Accessed: 15 April 2023*	#1	(“Lower Extremity”[MH] OR “Anterior Cruciate Ligament”[MH] OR “Kinetics”[MH] OR kinetic*[tiab] OR ground-reaction-force*[tiab] OR ground-reaction-moment*[tiab] OR ground-reaction-torq*[tiab] OR joint dynamic*[tiab] OR joint moment*[tiab] OR joint load*[tiab] OR joint torque[tiab] OR joint force*[tiab] OR joint mechanic*[tiab])
	#2	(((wearable*[tiab] OR body-worn*[tiab]) AND (electronic*[tiab] OR device*[tiab] OR sensor*[tiab] OR motion*[tiab])) OR ambulatory-monitor*[tiab] OR IMU[tiab] OR IMMU[tiab] OR MIMU[tiab] OR inertial-sensor*[tiab] OR inertial-device*[tiab] OR inertial-unit*[tiab] OR inertial-data*[tiab] OR inertial-motion*[tiab] OR motion-capture*[tiab] OR motion-sensor*[tiab] OR inertial-measurement-unit*[tiab] OR ((wireless*[tiab] OR inertial*[tiab]) AND (capture*[tiab])) OR ((3D[tiab] OR 3-d[tiab] OR 3-dimen*[tiab] OR threedimension*[tiab] OR three-dimension*[tiab]) AND (sensor*[tiab] OR motion*[tiab])) OR 3D analys*[tiab] OR 3-dimensional-analys*[tiab] OR threedimensional-analys*[tiab] OR three-dimensional-analys*[tiab] OR accelerometer*[tiab] OR accelero-meter*[tiab])
	#3	(algorithm*[tiab] OR algorythm*[tiab] OR early-ident*[tiab] OR AI[tiab] OR automated-reason*[tiab] OR nearest-neighbo*[tiab] OR random-forest*[tiab] OR machine-learn*[tiab] OR deep-learn*[tiab] OR supervised-learn*[tiab] OR unsupervised-learn*[tiab] OR predict*[tiab] OR estimat*[tiab] OR comput*[tiab] OR calculate*[tiab] OR Anybody [tiab] OR Opensim [tiab] OR bayes-network*[tiab] OR calibration*[tiab] OR crossvalidat*[tiab] OR validat*[tiab] OR artificial-intellig*[tiab] OR artificial-intellig*[tiab] OR artificial-network*[tiab] OR machine-intellig*[tiab] OR neural-network*[tiab])
	#4	NOT ((“Review Literature as Topic”[MH] OR “Review” [Publication Type] OR “Systematic Review” [Publication Type] OR systematic-review*[ti]))
**SPORTDiscus** [Filters—language: English; document type: article Expanders—apply related words; apply equivalent subjects Search modes—Boolean/phrase] *Last Accessed:23 March 2022*	#1	((DE “ANTERIOR cruciate ligament” OR (DE “LEG” OR DE “ANKLE” OR DE “FOOT” OR DE “KNEE”) OR (DE “DYNAMICS” OR DE “BODY movement” OR DE “MOTION” OR DE “BODY movement” OR DE “PHYSICS”)) OR (TI(kinetic* OR ((ground*) N2 (reaction*) N2 (force* OR moment* OR torque*)) OR ((ankle* OR foot OR feet OR leg OR legs OR knee OR knees OR thigh* OR lower-leg* OR joint OR joints) N3 (force* OR moment* OR torque* OR dynamic* OR load* OR mechanic*)) OR (AB (kinetic* OR ((ground*) N2 (reaction*) N2 (force* OR moment* OR torque*)) OR ((ankle* OR foot OR feet OR leg OR legs OR knee OR knees OR thigh* OR lower-leg* OR joint OR joints) N3 (force* OR moment* OR torque* OR dynamic* OR load* OR mechanic*))
	#2	(TI (((wearable* OR body-worn*) N3 (electronic* OR device* OR sensor* OR motion*)) OR ((wireless* OR inertial* OR IMU OR IMMU OR MIMU) N3 (sensor* OR device* OR unit* OR data OR capture* OR analys*)) OR ((ambulat*) N3 (monitor*)) OR ((3D OR 3-d OR 3-dimen* OR threedimension* OR three-dimension*) N3 (sensor* OR motion* OR analys*)) OR accelerometer* OR accelero-meter*)) OR (AB(((wearable* OR body-worn*) N3 (electronic* OR device* OR sensor* OR motion*)) OR ((wireless* OR inertial* OR IMU OR IMMU OR MIMU) N3 (sensor* OR device* OR unit* OR data OR capture* OR analys*)) OR ((ambulat*) N3 (monitor*)) OR ((3D OR 3-d OR 3-dimen* OR threedimension* OR three-dimension*) N3 (sensor* OR motion* OR analys*)) OR accelerometer* OR accelero-meter*)))
	#3	(TI (algorithm* OR algorythm* OR AI OR automated-reason* OR computer-heurist* OR ((bayes*) N3 (combiner* OR network*)) OR ((nearest) N3 (neighbo*)) OR ((random*) N3 (forest*)) OR ((support*) N6 (vector*) N6 (machine* OR active)) OR ((extract*) N6 (feature*) N6 (vector*)) OR ((artificial* OR ambient* OR machine* OR algorithm* OR neural) N3 (intelligen* OR network*)) OR ((automat*) N3 (pattern*) N3 (recogni*)) OR ((learn*) N3 (algori*)) OR ((machine* OR deep OR supervis* OR unsupervis* OR bayes) N3 (learn*)) OR predict* OR estimat* OR comput* OR calculate* OR Anybody OR Opensim OR calibration* OR crossvalidat* OR validat* OR ((probab* OR predict*) N3 (system* OR model* OR score* OR scoring*)))) OR (AB(algorithm* OR algorythm* OR AI OR automated-reason* OR computer-heurist* OR ((bayes*) N3 (combiner* OR network*)) OR ((nearest) N3 (neighbo*)) OR ((random*) N3 (forest*)) OR ((support*) N6 (vector*) N6 (machine* OR active)) OR ((extract*) N6 (feature*) N6 (vector*)) OR ((artificial* OR ambient* OR machine* OR algorithm* OR neural) N3 (intelligen* OR network*)) OR ((automat*) N3 (pattern*) N3 (recogni*)) OR ((learn*) N3 (algori*)) OR ((machine* OR deep OR supervis* OR unsupervis* OR bayes) N3 (learn*)) OR predict* OR estimat* OR comput* OR calculate* OR Anybody OR Opensim OR calibration* OR crossvalidat* OR validat* OR ((probab* OR predict*) N3 (system* OR model* OR score* OR scoring*)))))

Note: All search queries with multiple successive searches (indicated with #) were combined with the “AND” operator.
