# Supplementary material for: Estimation of Kinetics Using IMUs to Monitor and Aid in Clinical Decision-Making during ACL Rehabilitation: A Systematic Review
Source: Sensors (Basel). 2024 Mar 28;24(7):2163. doi: 10.3390/s24072163 (PMC11014074; doi:10.3390/s24072163)
Supplement: Supplementary file 1 [file sensors-24-02163-s001.zip › Supplementary_Accuracy Tables (S3,S4,S5,S6).pdf]

Table S3: GRF and GRM estimation accuracies of the included articles

Ref. No.	Model	Activity/ Method	RMSE	RMSE%	rRMSE [%]	NRMSE (%)	Correlation coefficient	MAD	MAE	MAD [%]
[1]	BM	Normal walk		AP: 4.8±0.7; M-L: 4.8±0.5; V: 6.5±1.2			AP: 0.89±0.1; M-L: 0.55±0.1; V:0.80±0.1			
		L walk		AP: 7.3±1.5; M-L: 6.6±0.8; V: 7.2±1.7			AP: 0.84±0.1; M-L: 0.45±0.1; V:0.89±0.1			
		Slow walk		AP: 5.1±1.1; M-L: 4.2±0.8; V: 4.6±1.7			AP: 0.72±0.2; M-L: 0.51±0.2; V:0.55±0.2			
		Walk and turn		AP: 5.6±1.4; M-L: 5.5±0.8; V: 5.3±1.2			AP: 0.81±0.1; M-L: 0.37±0.2; V:0.85±0.1			
		Slalom walk		AP: 7.4±2.3; M-L: 7.4±1.4; V: 6.8±1.7			AP: 0.70±0.1; M-L: 0.48±0.1; V:0.82±0.1			
		Asymmetrical walk		AP: 6.5±1.7; M-L: 5.9±1.0; V: 5.7±1.2			AP: 0.56±0.3; M-L: 0.30±0.3; V:0.75±0.2			
[2]	ML-RCN [LSTM with foot strike and sacral+ right foot accelerometer]	Running	GRF: 0.16±0.04		GRF: 6.4±1.5					
	ML-RCN [LSTM without foot strike and only sacral accelerometer]		GRF: 0.17±0.05		GRF: 6.7±1.7					
[3]	BM	Running (2m/s)	GRF: 0.45#							
		Running (3m/s)	GRF: 0.47#							
		Running (4m/s)	GRF: 0.53#							

		Running (5m/s)	GRF: 0.59#				
[4]	ML	Walking		~5			
[5]	ML: Single subject training and evaluation	Running	VGRF: 0.09±0.02 to 0.33±0.08				VGRF: 0.95-0.99
	ML: Multiple subject training		VGRF: 0.16±0.03 to 0.60±0.09				VGRF: 0.90-0.99
[6]	BM	Acceleration	GRF: 2.82±0.7	GRF: 8.4±14			
		Decelerations	GRF: 5.77±1.8	GRF: 6.1±8.8			
		90° Cuts	GRF: 2.67±0.7	GRF: 3.3±4.1			
		Running (2-3m/s)	GRF: 1.62±0.4	GRF: 1.8±2.0			
		Running (4-5m/s)	GRF: 2.48±0.6	GRF: 3.1±5.7			
		Running (>6m/s)	GRF: 4.35±1.3	GRF: 6.4±7.6			
		All tasks averaged	GRF: 3.26±1.7	GRF: 4.8±8.3			
[7]	ML	Walking				VGRF: 5.37-8.54	
[8]	ML	Walking				AP: 7.00±0.80; M-L: 9.00±2.30; V: 5.00±1.10	
[9]	BM	Walking				VGRF: 4-8	
[10]	Statistical	Walking		AP-Healthy: 4.62; AP-Patient: 2.64			
[11]	MS	Jogging	VGRF: 0.074-0.106				
		Skipping	VGRF: 0.087-0.121				
		Left-leg single support	VGRF: 0.02-0.068				

		Left-leg single support jump	VGRF: 0.140-0.215		
		Side to side Jump	VGRF: 0.108-0.274		
		All tasks averaged	VGRF: 0.112		
[12]	BM	cadence 60 steps/min	H: 0.043±0.010 V: 0.23±0.054		
		cadence 80 steps/min	H: 0.056±0.017 V: 0.27±0.028		
		cadence 100 steps/min	H: 0.059±0.042 V: 0.28±0.044		
		cadence 120 steps/min	H: 0.076±0.031 V: 0.31±0.012		
[13]			3DGRF: <0.2		
[14]	ML	Running: 8km/h	VGRF: 0.017±0.010		VGRF: 0.99a
		Running: 9km/h	VGRF: 0.015±0.0072		VGRF: 0.99a
		Running: 10km/h	VGRF: 0.017±0.0091		VGRF: 0.99a
[15]	BM	Normal walk		AP: 4.47±1.42; M-L: 4.38±1.17; V: 5.14±0.89;	AP: 0.68±0.24; M-L: 0.29±0.21; V: 0.73±0.11;
		L walk		AP: 5.42±1.35; M-L: 4.86±1.04; V: 5.02±0.85;	AP: 0.66±0.14; M-L: 0.40±0.21; V: 0.81±0.06;
		Walk and turn		AP: 5.55±1.50; M-L: 4.72±2.16; V: 5.46±1.46;	AP: 0.64±0.25; M-L: 0.46±0.34; V: 0.79±0.05;
		Walk and turn twice		AP: 6.92±1.89; M-L: 5.02±1.33; V: 5.54±0.84;	AP: 0.61±0.19; M-L: 0.50±0.16; V: 0.82±0.04;

		Slalom walk	AP: 5.50±0.94;<br>M-L: 5.00±0.57;<br>V: 4.38±1.13;		AP: 0.56±0.08;<br>M-L: 0.43±0.05;<br>V: 0.81±0.04;		
[16]	BM	Ski jump and take-off					deviation %<br>GRF: 12
[17]	ML	Walking: Slow		AP: 6.49 ± 2.51;<br>V: 6.80 ± 2.85;			
		Walking: Moderate		AP: 6.16 ± 1.76;<br>V: 6.26 ± 1.24;			
		Walking: Fast		AP: 6.70 ± 2.99;<br>V: 8.21 ± 3.63;			
[18]	BM	Slow walk	AP: 15.00±5.60;<br>V: 11.00±3.80;				
		Normal walk	AP: 12.30±4.00;<br>V: 11.30±4.30;				
		Fast walk	AP: 10.80±2.30;<br>V: 10.10±2.50;				
		Asymmetrical walk with smaller right step	AP: 24.70±14.80;<br>V: 13.10±6.40;				
		Asymmetrical walk with smaller left step	AP: 15.30±4.10;<br>V: 11.10±5.00;				
		Total	AP: 15.60±9.00;<br>V: 11.30±4.70;				
[19]	ML	Walking			AP:0.97; M-L: 0.98; V:0.80	AP: 1.8 ± 0.3; M-L: 1.4 ± 0.3; V: 4.5 ± 1.1;	AP: 4.60 ± 0.70;<br>M-L: 10.50 ± 3.30; V: 4.00 ± 0.80;
[20]	ML-3MLP	Walking			AP:0.97; M-L: 0.80; V:0.97	AP:1.83 ± 0.68;<br>M-L: 1.35 ± 0.46;<br>V: 5.10 ± 1.64;	AP: 5.20 ± 2.00;<br>M-L: 12.80 ± 5.60; V: 4.70 ± 1.40;

	ML-1MLP				AP:0.97; M-L: 0.80; V:0.97	AP: 1.90 ± 0.60; M-L: 1.40 ± 0.50; V: 5.20 ± 1.70;	AP: 5.40 ± 1.80; M-L: 13.00 ± 6.10; V: 4.80 ± 1.50;
[21]	ML	walking 0.7m/s		AP: 13.52 ± 4.38; M-L: 17.75 ± 6.41; V: 6.86 ± 2.21;			
		walking 1 m/s		AP: 8.31 ± 2.89; M-L: 15.23± 5.50; V: 6.17 ± 1.81;			
		walking 1.3 m/s		AP: 7.34 ± 2.46; M-L: 14.19 ± 3.91; V: 6.08 ± 1.88;			
		walking 1.6 m/s		AP: 7.67 ± 2.73; M-L: 15.04 ± 2.78; V: 7.68 ± 3.15;			
		Total		AP: 9.21 ± 4.06; M-L: 15.55 ± 5.03; V: 6.70 ± 2.41;			
[22]	BM	Squats and STS	H: 10N; V: 15N;				
[23]	ML	Walking slow		AP: 5.40 ± 1.31; V: 5.82 ± 0.58	AP: 0.98 ± 0.01; V: 0.98 ± 0.01		
		Walking self-selected		AP: 5.23 ± 0.91; V: 5.86 ± 0.63	AP: 0.98 ± 0.01; V: 0.98 ± 0.01		
		Walking fast		AP: 5.20 ± 0.83; V: 7.22 ± 1.88	AP: 0.98 ± 0.01; V: 0.98 ± 0.01		
		Total		AP:5.28 ± 0.99; V: 6.30 ± 1.31	AP: 0.98 ± 0.01; V: 0.98 ± 0.01		

[24]	MS	Walking		AP: 5.50±1.20; M-L: 2.10±0.60; V: 9.30±3.00; FGRM: 0.90±0.60; SGRM: 1.60±0.60; TGRM: 0.20±0.10	AP: 15.00±2.40; M-L: 18.50±3.20; V: 7.70±2.10; FGRM: 38.00±23.10; SGRM: 17.50±6.80; TGRM: 23.30±7.20	AP:0.91; M-L: 0.80; V:0.97; FGRM:0.64; SGRM:0.91; TGRM:0.82
[25]	BM	Walking Normal	AP: 0.03±0.007; M-L: 0.02±0.003; V: 0.06±0.035; FGRM: 0.01±0.004; SGRM: 0.01±0.004; TGRM: 0.003±0.001		AP: 9.40±2.50; M-L: 13.10±2.80; V: 5.30±3.10; FGRM: 29.60±9.30; SGRM: 12.40±3.40; TGRM: 18.20±4.70	AP: 0.97; M-L: 0.86; V: 0.99; FGRM: 0.71; SGRM: 0.93; TGRM: 0.83
		Walking slow	AP: 0.04±0.012; M-L: 0.02±0.005; V: 0.08±0.039; FGRM: 0.01±0.004; SGRM: 0.02±0.006; TGRM: 0.003±0.001		AP: 10.40±3.20; M-L: 13.80±3.30; V: 6.30±3.30; FGRM: 30.20±9.30; SGRM: 13.30±3.80; TGRM: 18.80±4.80	AP: 0.96; M-L: 0.85; V: 0.99; FGRM: 0.71; SGRM: 0.92; TGRM: 0.81
		Walking fast	AP: 0.05±0.011; M-L: 0.02±0.004; V: 0.09±0.040; FGRM: 0.01±0.004; SGRM: 0.02±0.005; TGRM: 0.004±0.001		AP: 10.90±3.10; M-L: 14.60±3.10; V: 6.90±3.00; FGRM: 30.60±8.00; SGRM: 16.10±3.20; TGRM: 21.60±4.20	AP: 0.95; M-L:0.82; V:0.99; FGRM: 0.71; SGRM: 0.84; TGRM: 0.75
[26]	ML-CaffeNet	Running and side stepping				AP: 0.90± 0.05; M-L: 0.87± 0.09; v: 0.89± 0.09; FGRM:

					0.69±0.40;<br>SGRM:<br>0.55±0.46;<br>TGRM: 0.71±0.25
	ML-ResNet-50				AP: 0.90± 0.08;<br>M-L: 0.87± 0.10;<br>v: 0.85± 0.13;<br>FGRM:<br>0.64±0.42;<br>SGRM:<br>0.62±0.45;<br>TGRM:<br>0.70±0.28;
[27]	ML	Walking 0.4m/s	VGRF: 0.12 ±0.03	VGRF: 9.40±2.81	VGRF: 0.94 ± 0.01
		Walking 0.7m/s	VGRF: 0.12 ± 0.04	VGRF: 8.87±3.45	VGRF: 0.94 ± 0.03
		Walking 1.0m/s	VGRF: 0.11 ±0.03	VGRF: 8.58±2.62	VGRF: 0.95 ± 0.02
		Walking 1.3m/s	VGRF: 0.11±0.03	VGRF: 8.24±2.39	VGRF: 0.96 ± 0.02
		Walking 1.6m/s	VGRF: 0.13±0.02	VGRF: 9.38 ± 2.52	VGRF: 0.95 ± 0.02
[28]	ML	Unilateral landing (combined flight+ ground)	GRF: 0.42		GRF: 0.8
		Bilateral landing (combined flight+ ground	GRF: 0.39		GRF: 0.92
[29]	BM	Standing sprint start	AP: 400.05 ± 219.66;<br>M-L: 406.73 ± 260.82; V: 368.15 ± 210.70*		AP: 0.64 ± 0.15;<br>M-L: -0.24 ± 0.31;<br>V: 0.50 ± 0.30[b]

		Change of direction task	AP: 609.60 ± 185.24; M-L: 551.48 ± 199.56; V: 423.12 ± 171.80 *				AP: 0.51 ± 0.22; M-L: 0.13 ± 0.25; V: 0.66 ± 0.23b
[30]	ML	Outdoor controlled walking			vGRF: 3.8		
		Outdoor free walking			vGRF: <5		
[31]	BM	Walking	73.44 (Max error 160.20) *				0.952
[32]	MS	Walking		AP: 4.1± 1.2; V: 11.1± 3.4	AP: 9.7±2.1; V:9.6±2.5		AP: 0.95; V: 0.95
		Running		AP: 10.7± 3.9; V: 32.0± 7.9	AP: 13.5±4.2; V:12.8 ±3.6		AP: 0.94; V: 0.94
[33]	BM	Walking	AP: 53.71±25.86; M-L: 39.23±8.67; V: 37.14±14.04;		AP: 51.68±11.22; M-L:60.61±12.82; V: 3.57±0.74;		AP: 0.58; M-L: 0.91; V: 0.98[c];
		Jumping	AP: 98.42±30.54; M-L: 111.43±37.58; V: 126.19±55.21;		AP: 182.59±122.08; M-L: 26.62±5.84; V: 6.08±2.07;		AP: 0.79; M-L: 0.62; V: 0.95[c];
		Lifting	AP: 60.99±21.25; M-L: 88.27±22.13; V: 84.04±42.99[*]		AP: 73.11±25.00; M-L: 141.31±58.21; V: 8.31±5.05		AP: -0.75; M-L: -0.05; V: 0.92[c];
[34]	ML	Walking and running			AP: 2; M-L: 6;		
[35]	ML	Walking and running				VGRF<5	
[36]	BM	Alternate lateral jump 1.2m	M-L: 55.93; V: 43.42*			M-L: 11.98; V:4.45;	

		Alternate lateral jump 1.4m	M-L: 65.66; V: 52.17*			M-L: 12.75; V: 5.23;				
		Alternate lateral jump 1.6m	M-L: 65.22; V: 76.21*			M-L: 12.10; V: 7.51;				
		Alternate lateral jump 1.8m	M-L: 57.35; V: 71.59*			M-L: 10.59; V: 7.10;				
[37]	Statistical	Countermovement Jump in water					VGRF: 0.7453[d]			
[38]	ML- Data filtered @12Hz	Vertical drop Jump	AP: 0.0409; M-L: 0.029; V: 0.0433;							
	ML- Data filtered @24Hz		AP: 0.0188; M-L: 0.0087; V: 0.0381;							
	ML- Data filtered @32Hz		AP: 0.0184; M-L: 0.0081; V: 0.035;							
[39]	BM	Walking and swaying								% diff DLF=3%
[40]	BM	Squat				VGRF: 4.3±2.4	VGRF: 0.98±0.02[a]			
[41]	ML	Treadmill walking				AP: 2.86 ± 1.01; M-L: 4.12 ± 1.94; V: 4.42 ± 2.91; 2nd Dataset: AP: 6.42 ± 0.5; M-L: 7.49 ± 1.09; V: 7.86 ± 1.1;	AP: 0.967±0.031; M-L: 0.917±0.044; V: 0.977±0.037; 2nd Dataset: AP: 0.917 ± 0.014; M-L: 0.885 ± 0.02; V: 0.959 ± 0.011;			
		Overground walking				AP: 3.77 ± 1.30; M-L: 8.65 ± 1.34; V:5.49 ± 4.36;	AP: 0.922±0.078; M-L: 0.826±0.095; V: 0.957±0.088;			
		Ramp				AP: 3.46 ± 0.94; M-L: 3.12 ± 0.99; V: 4.34 ± 2.81;	AP: 0.952±0.040; M-L: 0.856±0.061; V: 0.979±0.042;			

		Stair				AP: 2.56 ± 0.43; M-L: 3.07 ± 0.88; V: 4.05 ± 1.88;	AP: 0.927±0.029; M-L: 0.884±0.043; V: 0.984±0.024;			
[42]	ML	Running				Stance GRF: 0.189-0.228; GRF: 0.191-0.309				
[43]	ML	Walking and running		VGRF: 6.8 ± 0.3						
[44]	ML-cycle duration	Walking							Healthy: AP: 0.032; M-L: 0.016; V: 0.123; MKOA: AP: 0.025; M-L: 0.014; V: 0.107;	
	ML-Stance proportion								Healthy: AP: 0.032; M-L: 0.016; V: 0.126; MKOA: AP: 0.028; M-L: 0.014; V: 0.112;	
	ML-Stance duration								Healthy: AP: 0.031; M-L: 0.016; V: 0.125; MKOA: AP: 0.026; M-L: 0.014; V: 0.105;	
	ML-Standard training								Healthy: AP: 0.035; M-L: 0.016; V: 0.134; MKOA: AP: 0.031; M-L: 0.016; V: 0.132;	
[45]	BM-Sacrum	vertical countermovement jump	VGRF: 14N							
	BM-Upper back		VGRF: 27N							
	BM-Chest		VGRF: 67N							
	BM-Combined		VGRF: 20N							

AP- Anterior-posterior, M-L-Medio-Lateral, V-Vertical, H-Horizontal, FGRM- Frontal GRM, SGRM: Sagittal GRM, TGRM: Transverse GRM, all coefficients reported are Pearson correlation coefficients except when marked with a, b, c, d. a- Cross correlation coefficient, b-Pearson product moment correlation, c-Inter class correlation coefficient, d-Lin's concordance correlation coefficient. #- Median RMSE

Table S4: Peak GRF and peak VGRF estimation accuracies

Ref. No.	Method	Activity/Method	Average absolute difference[N]	Average absolute percentage difference [%]	RMSE	nRMSE [%]	rRMSE %	Error %	MAE	MAPE	Correlation
[46]	Statistical	No load walking	pVGRF: 49.9±24.8*	pVGRF: 5.3±2.9							
		Walking with 14kg	pVGRF: 49.6±15.9*	pVGRF: 4.4±1.5							
		Walking with 27kg	pVGRF: 56.7±21.3*	pVGRF: 4.5±1.7							
		Walking with 34kg	N/A	N/A							
		Walking with46kg	pVGRF: 63.3±43.5*	pVGRF: 4.6±3.0							
[47]	BM	Running (9km/h)			pVGRF: 0.13		pVGRF: 5.3				
		Running (11km/h)			pVGRF: 0.13		pVGRF: 5.1				
		Running (13km/h)			pVGRF: 0.19		pVGRF: 7.4				
[48]	Statistical (Regression: Mixed model)			5.2±1.6							
	Statistical (Regression: Generalized model)			9±4.2							
[49]	BM	Running 0°						pVGRF: 11.7			pVGRF:0.76[e]
		Running 45°						pVGRF: 14.5			pVGRF: 0.67[e]
		Running 90°						pVGRF: 17.2			pVGRF: 0.47[e]
		Running 180°						pVGRF: 23.9			pVGRF: 0.23[e]

[5]	ML: Single subject training and evaluation	Running	pVGRF: <0.09								pVGRF: > 0.96
	ML: Multiple training and evaluation										
[50]	Statistical: Lower back	Walking			pGRF: 81.5; pVGRF: 104.1				pGRF: 62.1; pVGRF: 76.3*	pGRF: 5.5; pVGRF: 6.6	
	Statistical: Hip				pGRF: 74.1; pVGRF: 86.3				pGRF: 57.4; pVGRF: 62.5*	pGRF: 5.4; pVGRF: 5.7	
[51]	Statistical	Walking (various speeds)			pGRF:0.076				pGRF: 5.98%		
[52]	BM	Running-slow			pVGRF: 0.19±0.04						N/A
		Running-comfortable			pVGRF: 0.18±0.04						0.95[e]
		Running-fast			pVGRF: 0.19±0.05						N/A
[53]	ML	Double limb jump and landing			pVGRF:0.22 ± 0.002	pVGRF: 6.0 ± 0.05					
[54]	ML-QRF	Running			pVGRF: 0.150					pVGRF: 4.27 ± 2.85	
	ML-LR				pVGRF: 0.139					pVGRF: 4.04 ± 2.57	
[55]	Statistical	Walking			pVGRF: 75.6*				pVGRF: 59.00*	pVGRF: 6.00	
		Running			pVGRF: 170.3*				pVGRF: 132.5*	pVGRF: 8.01	
		Walking & running combined			pVGRF: 133*				pVGRF: 96.8*	pVGRF: 7.10	
[56]	Statistical-Lower back	Drop jump, box jump and			pVGRF: 451.3 ± 509.8; pGRF: 456.1 ± 508.5*				pVGRF: 371.0 ± 257.1; pGRF: 376.3 ± 257.9*	pVGRF: 14.4 ± 10.9; pGRF: 14.5 ± 10.7	

	Statistical- Hip	continuous jumps	pVGRF: 422.9 ± 578.1; pGRF: 396.6 ± 549.2*	pVGRF: 322.9 ± 273.3; pGRF: 302.1 ± 257.2*	pVGRF: 13.3 ± 15.0; pGRF: 12.3 ± 13.4
	Statistical- Ankle		pVGRF: 450.1 ± 581.0; pGRF: 438.2 ± 569.4*	pVGRF: 350.4 ± 282.8; pGRF: 341.2 ± 275.1*	pVGRF: 14.4 ± 14.5; pGRF: 13.9 ± 13.4
[45]	BM-Sacrum	vertical countermovement jump	pVGRF: 88N*		
	BM-Upper back		pVGRF: 150N*		
	BM-Chest		pVGRF: 360N*		
	BM-Combined		pVGRF: 100N*		
[57]	ML- LR	Running	pVGRF: 0.12		3.7 ± 3.0
	ML-SVR		pVGRF: 0.13		3.8 ± 3.1
	ML- NN2		pVGRF: 0.13		3.8 ±3.2

pVGRF-peak vertical GRF, pGRF-peak GRF, all reported values are accuracies normalized for bodyweight expect when marked with *, * -values not normalized for body weight, all coefficients reported are Pearson correlation coefficients except when marked with e, e- spearman correlation coefficient, N/A- Not available, BM: Biomechanical modelling, MS: Musculoskeletal modelling, ML-Machine Learning.

Table S5: Joint moment estimation accuracies of the included articles

Ref. No.	Method	Activity	Joint	Correlation coefficient	nRMSE [%]	RMSE	RMSE [%]	rRMSE
[58]	BM	STS	Hip	MLDK patients: 0.09 to 0.35;				
			Knee	MLDK patients:0.11 to 0.29;				
			Ankle	MLDK patients:0.03 to 0.35;				
[59]	ML(FFNN)	Walking	Hip	Ab/Ad: 0.98; F/E: 0.99; ER/IR: 0.99	Ab/Ad:10.29; F/E: 7.34; ER/IR: 6.50			
			Knee	Ab/Ad: 0.98; F/E: 0.98; ER/IR: 0.88	Ab/Ad:9.46; F/E: 10.58; ER/IR: 17.12			
			Ankle	Ab/Ad: 0.99; F/E: 0.92; ER/IR: 0.93	Ab/Ad: 7.39; F/E: 22.60; ER/IR: 17.59			
	ML(LSTM)		Hip	Ab/Ad: 0.99; F/E: 0.98; ER/IR: 0.99	Ab/Ad: 9.83; F/E: 8.34; ER/IR: 8.64			
			Knee	EV: 0.96; PF/DF: 0.96; Ax: 0.86	EV: 11.85; PF/DF: 14.52; Ax: 20.05			
			Ankle	EV: 0.99; PF/DF: 0.90; Ax: 0.94	EV: 7.32; PF/DF: 24.19; Ax: 19.68			
[60]	ML (TCN)	Hip steady state	Hip			0.13 ± 0.02		
		Hip ambulation mode transitions				0.15 ± 0.03		
	ML(FCNN)	Hip steady state				0.15 ± 0.02		
		Hip ambulation mode transitions				0.17 ± 0.04		
	ML(LSTM)	Hip steady state				0.15 ± 0.02		
		Hip ambulation				0.17 ± 0.03		

		mode transitions						
[61]	Biomechanical	Walking and running	Hip				~20 (error %)	
[12]	BM	cadence 60 steps/min	Hip			0.81±0.27		
		cadence 80 steps/min				1.02±0.69		
		cadence 100 steps/min				1.03±0.67		
		cadence 120 steps/min				0.96±0.59		
		cadence 60 steps/min	Knee			0.57±0.08		
		cadence 80 steps/min				0.55±0.25		
		cadence 100 steps/min				0.54±0.22		
		cadence 120 steps/min				0.58±0.23		
[13]	BM	Standing balance	L5/S1, Hip, ankle			SG and C averaged < 0.016		
[62]	ML	Walking	Hip, Knee, ankle		>13			
[17]	ML	Walking: Slow	Hip		11.67 ± 2.03			
		Walking: Moderate			10.74 ± 1.26			
		Walking: Fast			10.65 ± 1.06			
		Walking: Slow	Knee		10.58 ± 1.53			
		Walking: Moderate			9.63 ± 1.40			
		Walking: Fast			9.33 ± 3.42			
		Walking: Slow	Ankle		9.63 ± 3.12			
		Walking: Moderate			9.24 ± 1.91			
		Walking: Fast			9.37 ± 1.68			

[18]	BM	Fast walk	Hip		14.80±4.60
		Asymmetrical walk with smaller right step			34.50±14.10
		Asymmetrical walk with smaller left step			20.50±8.20
		Total			23.40±11.30
		Slow walk	Knee		29.00±12.30
		Normal walk			25.80±11.20
		Fast walk			21.40±8.50
		Asymmetrical walk with smaller right step			34.40±15.50
		Asymmetrical walk with smaller left step			34.20±15.60
		Total			29.00±13.70
		Slow walk	Ankle		21.30±9.10
		Normal walk			18.60±7.20
		Fast walk			17.10±6.70
		Asymmetrical walk with smaller right step			24.50±11.10
		Asymmetrical walk with smaller left step			20.40±8.80
		Total			20.40±9.00
[21]	ML	walking 0.7m/s	Hip	Ab/Ad: 15.38 ± 4.13; F/E:16.08 ± 6.37; ER/IR: 23.66 ± 13.43;	

		walking 1 m/s			Ab/Ad: 12.04 ± 4.17; F/E:11.16 ± 4.64; ER/IR: 17.12 ± 8.45;		
		walking 1.3 m/s			Ab/Ad: 11.50 ± 3.84; F/E: 9.29 ± 3.10; ER/IR: 14.47 ± 6.50;		
		walking 1.6 m/s			Ab/Ad: 12.43 ± 4.20; F/E: 9.08 ± 2.44; ER/IR: 13.72 ± 5.85;		
		All speeds averaged			Ab/Ad: 12.84 ± 4.35; F/E: 11.40 ± 5.22; ER/IR:17.24 ± 9.85;		
		walking 0.7m/s	Knee		Ab/Ad: 20.96 ± 9.40; F/E: 33.64 ± 17.11; ER/IR: 28.62 ± 12.59;		
		walking 1 m/s			Ab/Ad: 16.88 ± 7.76; F/E: 25.77 ± 14.14; ER/IR:22.69 ± 13.33;		
		walking 1.3 m/s			Ab/Ad: 15.95 ± 10.49; F/E:19.46 ± 9.12; ER/IR: 18.56 ± 8.19;		
		walking 1.6 m/s			Ab/Ad: 16.33 ± 10.18; F/E: 17.47 ± 7.26; ER/IR:16.16 ± 6.51;		
		All speeds averaged			Ab/Ad: 17.53 ± 9.70; F/E: 24.08 ± 14.01; ER/IR: 21.51 ± 11.54;		
		walking 0.7m/s	Ankle		PF/DF: 18.20 ± 9.57;		
		walking 1 m/s			PF/DF: 12.80 ± 6.78;		
		walking 1.3 m/s			PF/DF: 11.54 ± 5.65;		
		walking 1.6 m/s			PF/DF: 11.78 ± 5.59;		
		All speeds averaged			PF/DF: 13.58 ± 7.57;		
[63]	MS	Stair up	Knee	0.86		Ab/Ad: 0.01 ± 0.003	
		Stair down		0.74		Ab/Ad: 0.01 ± 0.005	

		Sit to stand		0.98		Ab/Ad: 0.01 ± 0.002		
[22]	BM	Squats and STS	Hip, knee, ankle	0.80-0.98		0.01		
[24]	MS	Walking	Hip	Ab/Ad: 0.83; F/E: 0.92; ER/IR: 0.50			Ab/Ad: 1.40±0.70; F/E: 2.20±0.60; ER/IR: 0.50±0.20	Ab/Ad: 19.70±5.80; F/E: 19.40±4.20; ER/IR: 31.60±6.60
			Knee	Ab/Ad: 0.81; F/E: 0.58; ER/IR: 0.73			Ab/Ad: 1.10±0.40; F/E: 1.90±0.50; ER/IR: 0.30±0.10	Ab/Ad: 18.90±6.80; F/E: 29.80±7.60; ER/IR: 25.40±10.30
			Ankle	EV: 0.76; PF/DF: 0.93; Ax: 0.67			Ev: 0.60±0.20; PF/DF: 1.60±0.60; Ax: 0.50±0.20	Ev: 33.30±20.20; PF/DF: 15.10±6.60; Ax: 30.40±12.20
[31]	BM	Walking	Knee	0.737		8.77(Max error 25.61) *		
			Ankle	0.952		4.68 (Max error 11.29) *		
[32]	MS	Walking	Hip	0.76			1.5 ± 0.4	26.0 ± 9.80
			Knee	0.81			1.5 ± 0.4	27.10 ± 9.20
			Ankle	0.95			1.6±0.8	14.40±6.80
		Running	Hip	0.85			3.2 ±1.0	26.00 ±6.80
			Knee	0.94			3.4 ±1.2	16.70±7.10
			Ankle	0.96			3.2 ±2.1	17.10 ±10.8
[34]	ML	Walking and running	Knee, Ankle				6	
[53]	ML	Double limb jump and landing	Knee		pF/E: 10.6 ± 0.07	pF/E: 0.028 ± 0.0002		
[38]	ML- Data filtered @12Hz	Vertical drop Jump	Knee			Ab/Ad: 0.0302; F/E: 0.1159; ER/IR: 0.0114		
	ML- Data filtered @24Hz					Ab/Ad: 0.0334; F/E: 0.0816; ER/IR: 0.0121		
	ML- Data filtered @32Hz					Ab/Ad: 0.0295; F/E: 0.0748; ER/IR: 0.0117		
[41]	ML	Treadmill walking	Hip	Ab/Ad: 0.925 ± 0.046; F/E: 0.948±0.031	Ab/Ad: 6.03±2.28; F/E: 3.67±1.40			
		Overground walking		Ab/Ad: 0.810±0.131; F/E: 0.765±0.177	Ab/Ad: 8.16±2.70; F/E: 6.06±2.27			

Ramp		Ab/Ad: 0.899±0.061; F/E: 0.895±0.051	Ab/Ad: 5.69±2.09; F/E: 5.64±1.86
Stair		Ab/Ad: 0.922±0.038; F/E: 0.870±0.075	Ab/Ad: 4.51±1.55; F/E: 4.06±1.43
Treadmill walking	Knee	TKM: 0.917 ± 0.040; 2nd dataset: Ab/Ad: 0.805 ± 0.055; F/E: 0.865 ± 0.09	TKM: 3.25 ± 1.31; 2nd dataset: Ab/Ad: 8.27 ± 1.72; F/E:7.11 ± 1.47
Overground walking		TKM: 0.718 ± 0.191	TKM: 5.48 ± 1.97
Ramp		TKM: 0.912 ± 0.054	TKM: 5.05 ± 2.05
Stair		TKM: 0.928 ± 0.046	TKM: 4.35 ± 1.86
Treadmill walking	Ankle	TAM: 0.966 ± 0.038	TAM: 3.20 ± 2.01
Overground walking		TAM: 0.868 ± 0.117	TAM: 5.59 ± 2.49
Ramp		TAM: 0.930 ± 0.061	TAM: 4.35 ± 2.46
Stair		TAM: 0.944 ± 0.042	TAM: 3.96 ± 2.70

Ab/Ad: Abduction-adduction moment, F/E: Flexion-Extension moment, ER/IR: External-Internal rotation moment, pF/E: Peak Flexion-Extension moment, TKM: Total knee moment, PF/DF: Plantar flexion-Dorsi flexion, TAM- Total ankle moment, EV- Eversion-Inversion moment, AX- Axial moment, SG- Sagittal moment, C-Coronal moment, BM: Biomechanical modelling, MS: Musculoskeletal modelling, ML-Machine Learning

Table S6: Joint forces estimation accuracies of the included articles

Ref. No.	Method	Activity	Joint	Correlation coefficient	RMSE [N]	RMSE [%]	rRMSE [%]	Max error (N)	NRMSD	Difference %
[64]	BM	Walking	Heel	0.96-0.97	44.10-56.04			47.58-62.47		
			Phalange	0.95-0.97	59.85-66.22			44.74-67.01		
[65]	BM	Walking	Heel	0.95-0.99						
			Phalange	0.97-0.99						
[61]	BM	Walking and running	Hip							20-25
[66]	ML	Moderate running	Knee	AP: 0.90±0.30; M-L: 0.43±0.26; V: 0.94±0.33			AP: 18.90±5.50; M-L: 41.70±11.50; V: 14.20±4.00			pVKJF: 10.00±12.80
		Fast running		AP: 0.88±0.44; M-L: 0.42±0.41; V: 0.89±0.43			AP: 22.90±9.50; M-L: 43.40±12.00; V: 20.30±5.80			pVKJF: 16.10±34.20
		Running 90° clockwise turn		AP: 0.82±0.36; M-L: 0.38±0.35; V: 0.89±0.40			AP: 21.00±6.50; M-L: 36.70±18.40; V: 17.20±4.00			pVKJF: 17.40±36.30
		Running 90° counterclockwise turn		AP: 0.88±0.43; M-L: 0.37±0.42; V: 0.87±0.35			AP: 19.50±8.10; M-L: 37.20±11.50; V: 17.50±5.30			pVKJF: 19.30±28.00
		Left side cutting maneuver		AP: 0.86±0.41; M-L: 0.30±0.42; V: 0.86±0.44			AP: 22.00±7.30; M-L: 44.80±13.00; V: 19.40±6.60			pVKJF: 21.00±25.60
		Right-side cutting maneuver		AP: 0.84±0.35; M-L: 0.25±0.39; V: 0.86±0.39			AP: 21.50±5.20; M-L: 45.70±9.00; V: 19.00±5.40			pVKJF: 17.20±20.20
		Side shuffle cut		AP: 0.81±0.43; M-L: 0.35±0.45; V: 0.79±0.47			AP: 19.80±6.00; M-L: 36.50±9.30; V: 20.40±6.60			pVKJF: 2.60±19.30
		Walking		AP: 0.71±0.39; M-L: 0.60±0.31; V: 0.87±0.32			AP: 20.80±5.60; M-L: 27.70±5.70; V: 14.20±4.30			pVKJF: 13.80±16.20

		Walking 90° clockwise turn		AP: 0.65±0.31; M-L: 0.31±0.20; V: 0.81±0.27			AP: 23.00±6.20; M-L: 34.10±8.10; V: 16.90±4.50			pVKJF: 8.70±12.60
		Walking 90° counterclockwise turn		AP: 0.64±0.30; M-L: 0.48±0.34; V: 0.83±0.29			AP: 22.70±5.80; M-L: 29.10±6.00; V: 15.30±4.00			pVKJF: 19.50±24.50
		One-leg jump take-off		AP: 0.89±0.25; M-L: 0.31±0.46; V: 0.92±0.39			AP: 17.40±5.50; M-L: 45.90±19.70; V: 15.40±6.60			pVKJF: 8.00±18.70
		One-leg jump landing		AP: 0.77±0.53; M-L: 0.42±0.38; V: 0.84±0.43			AP: 25.10±9.40; M-L: 38.90±14.40; V: 16.70±7.20			pVKJF: 6.40±12.60
		Two-leg jump take-off		AP: 0.82±0.40; M-L: 0.51±0.23; V: 0.60±0.36			AP: 20.50±7.40; M-L: 27.80±2.90; V: 23.00±8.60			pVKJF: 60.80±59.80
		Two-leg jump landing		AP: 0.65±0.36; M-L: 0.54±0.32; V: 0.61±0.34			AP: 27.10±5.50; M-L: 37.60±6.80; V: 25.90±6.20			pVKJF: 22.90±34.70
		All tasks averaged		AP: 0.79±0.09; M-L: 0.40±0.10; V: 0.82±0.10			AP: 21.80±2.60; M-L: 38.00±6.10; V: 19.10±4.00			pVKJF: 17.00±13.60
[16]	BM	Ski jump (Take off phase)	Hip		43.5±92					11.2±13
			Knee		54.9±123					10.3±15
			Ankle		58.1±132					9.7±14
[63]	MS	Stair up	Knee	0.86	0.89±0.32					
		Stair down		0.85	0.90 ± 0.30					
		Sit to stand		0.92	0.40 ± 0.14					
[24]	MS	Walking	Hip	AP: 0.71; M-L: 0.73; PD: 0.78		AP: 17.60±7.60; M-L: 27.00±12.50; PD: 102.80±30.60	AP: 27.20±9.60; M-L: 23.00±7.40; PD: 21.70±4.50			
			Knee	AP: 0.82; M-L: 0.91; PD: 0.90		AP: 30.60±10.30; M-L: 12.00±3.50; PD: 63.10±26.90	AP: 25.80±9.70; M-L: 14.10±3.80; PD: 14.30±6.60			
			Ankle	AP: 0.84; M-L: 0.93; PD: 0.93		AP: 22.20±10.30; M-L: 24.30±8.90; PD: 88.50±30.60	AP: 26.10±10.20; M-L: 15.20±5.30; PD: 13.60±4.60			
[67]	BM		Ankle	H:0.63±0.20; V: 0.95±0.01	H: 11±4N; V: 19±8N				H: 130±45; V: 3±1.5	

pVKJF:-Peak Vertical Knee joint Force, AP- Anterior-posterior, M-L-Medio-Lateral, V-Vertical, H-Horizontal, PD-Proximodistal, BM: Biomechanical modelling, MS: Musculoskeletal modelling, ML-Machine Learning

References

1. Refai, M.I.M.; van Beijnum, B.F.; Buurke, J.H.; Veltink, P.H. Portable Gait Lab: Estimating 3D GRF Using a Pelvis IMU in a Foot IMU Defined Frame. *IEEE Trans Neural Syst Rehabil Eng* **2020**, *28*, 1308-1316, doi:10.1109/TNSRE.2020.2984809.
2. Alcantara, R.S.; Edwards, W.B.; Millet, G.Y.; Grabowski, A.M. Predicting continuous ground reaction forces from accelerometers during uphill and downhill running: a recurrent neural network solution. *PeerJ* **2022**, *10*, e12752, doi:10.7717/peerj.12752.
3. Nedergaard, N.J.; Verheul, J.; Drust, B.; Etchells, T.; Lisboa, P.; Robinson, M.A.; Vanrenterghem, J. The feasibility of predicting ground reaction forces during running from a trunk accelerometry driven mass-spring-damper model. *PeerJ* **2018**, *6*, e6105, doi:10.7717/peerj.6105.
4. Nagashima, M. Prediction of Plantar Forces During Gait Using Wearable Sensors and Deep Neural Networks. In Proceedings of the 41st Annual International Conference of the IEEE Engineering in Medicine and Biology Society (EMBC), Berlin, Germany, 2019; pp. 3629-3632.
5. Wouda, F.J.; Giuberti, M.; Bellusci, G.; Maartens, E.; Reenalda, J.; van Beijnum, B.-J.F.; Veltink, P.H. Estimation of Vertical Ground Reaction Forces and Sagittal Knee Kinematics During Running Using Three Inertial Sensors. *Frontiers in Physiology* **2018**, *9*, doi:10.3389/fphys.2018.00218.
6. Verheul, J.; Gregson, W.; Lisboa, P.; Vanrenterghem, J.; Robinson, M.A. Whole-body biomechanical load in running-based sports: The validity of estimating ground reaction forces from segmental accelerations. *J Sci Med Sport* **2019**, *22*, 716-722, doi:10.1016/j.jsams.2018.12.007.
7. Sharma, D.; Davidson, P.; Muller, P.; Piche, R. Indirect Estimation of Vertical Ground Reaction Force from a Body-Mounted INS/GPS Using Machine Learning. *Sensors (Basel)* **2021**, *21*, doi:10.3390/s21041553.
8. Shahabpoor, E.; Pavic, A.; Brownjohn, J.M.W.; Billings, S.A.; Guo, L.Z.; Bocian, M. Real-Life Measurement of Tri-Axial Walking Ground Reaction Forces Using Optimal Network of Wearable Inertial Measurement Units. *IEEE Trans Neural Syst Rehabil Eng* **2018**, *26*, 1243-1253, doi:10.1109/TNSRE.2018.2830976.
9. Shahabpoor, E.; Pavic, A. Estimation of vertical walking ground reaction force in real-life environments using single IMU sensor. *J Biomech* **2018**, *79*, 181-190, doi:10.1016/j.jbiomech.2018.08.015.
10. Revi, D.A.; Alvarez, A.M.; Walsh, C.J.; De Rossi, S.M.M.; Awad, L.N. Indirect measurement of anterior-posterior ground reaction forces using a minimal set of wearable inertial sensors: from healthy to hemiparetic walking. *J Neuroeng Rehabil* **2020**, *17*, 82, doi:10.1186/s12984-020-00700-7.
11. Recinos, E.; Abella, J.; Riyaz, S.; Demircan, E. Real-Time Vertical Ground Reaction Force Estimation in a Unified Simulation Framework Using Inertial Measurement Unit Sensors. *Robotics* **2020**, *9*, doi:10.3390/robotics9040088.
12. Ohtaki, Y.; Sagawa, K.; Inooka, H. A Method for Gait Analysis in a Daily Living Environment by Body-Mounted Instruments. *JSME International Journal Series C* **2001**, *44*, 1125-1132, doi:10.1299/jsmec.44.1125.
13. Noamani, A.; Nazarahari, M.; Lewicke, J.; Vette, A.H.; Rouhani, H. Validity of using wearable inertial sensors for assessing the dynamics of standing balance. *Med Eng Phys* **2020**, *77*, 53-59, doi:10.1016/j.medengphy.2019.10.018.
14. Ngoh, K.J.; Gouwanda, D.; Gopalai, A.A.; Chong, Y.Z. Estimation of vertical ground reaction force during running using neural network model and uniaxial accelerometer. *J Biomech* **2018**, *76*, 269-273, doi:10.1016/j.jbiomech.2018.06.006.
15. Mohamed Refai, M.I.; van Beijnum, B.F.; Buurke, J.H.; Veltink, P.H. Portable Gait Lab: Estimating Over-Ground 3D Ground Reaction Forces Using Only a Pelvis IMU. *Sensors (Basel)* **2020**, *20*, doi:10.3390/s20216363.
16. Logar, G.; Munih, M. Estimation of joint forces and moments for the in-run and take-off in ski jumping based on measurements with wearable inertial sensors. *Sensors (Basel)* **2015**, *15*, 11258-11276, doi:10.3390/s150511258.
17. Lim, H.; Kim, B.; Park, S. Prediction of Lower Limb Kinetics and Kinematics during Walking by a Single IMU on the Lower Back Using Machine Learning. *Sensors (Basel)* **2019**, *20*, doi:10.3390/s20010130.
18. Li, T.; Wang, L.; Yi, J.; Li, Q.; Liu, T. Reconstructing Walking Dynamics From Two Shank-Mounted Inertial Measurement Units. *IEEE/ASME Transactions on Mechatronics* **2021**, *26*, 3040-3050, doi:10.1109/tmech.2021.3051724.
19. Leporace, G.; Batista, L.A.; Nadal, J. Prediction of 3D ground reaction forces during gait based on accelerometer data. *Research on Biomedical Engineering* **2018**, *34*, 211-216, doi:10.1590/2446-4740.06817.
20. Leporace, G.; Batista, L.A.; Metsavaht, L.; Nadal, J. Residual analysis of ground reaction forces simulation during gait using neural networks with different configurations. In Proceedings of the 2015 37th Annual International Conference of the IEEE Engineering in Medicine and Biology Society (EMBC), Milan, Italy, 2015; pp. 2812-2815.

21. Lee, M.; Park, S. Estimation of Three-Dimensional Lower Limb Kinetics Data during Walking Using Machine Learning from a Single IMU Attached to the Sacrum. *Sensors (Basel)* **2020**, *20*, doi:10.3390/s20216277.
22. Kodama, J.; Watanabe, T. Examination of Inertial Sensor-Based Estimation Methods of Lower Limb Joint Moments and Ground Reaction Force: Results for Squat and Sit-to-Stand Movements in the Sagittal Plane. *Sensors (Basel)* **2016**, *16*, doi:10.3390/s16081209.
23. Kim, B.; Lim, H.; Park, S. Spring-loaded inverted pendulum modeling improves neural network estimation of ground reaction forces. *J Biomech* **2020**, *113*, 110069, doi:10.1016/j.jbiomech.2020.110069.
24. Karatsidis, A.; Jung, M.; Schepers, H.M.; Bellusci, G.; de Zee, M.; Veltink, P.H.; Andersen, M.S. Musculoskeletal model-based inverse dynamic analysis under ambulatory conditions using inertial motion capture. *Medical engineering & physics* **2019**, *65*, 68-77, doi:10.1016/j.medengphy.2018.12.021.
25. Karatsidis, A.; Bellusci, G.; Schepers, H.M.; de Zee, M.; Andersen, M.S.; Veltink, P.H. Estimation of Ground Reaction Forces and Moments During Gait Using Only Inertial Motion Capture. *Sensors (Basel)* **2016**, *17*, doi:10.3390/s17010075.
26. Johnson, W.R.; Mian, A.; Robinson, M.A.; Verheul, J.; Lloyd, D.G.; Alderson, J.A. Multidimensional Ground Reaction Forces and Moments From Wearable Sensor Accelerations via Deep Learning. *IEEE Trans Biomed Eng* **2021**, *68*, 289-297, doi:10.1109/TBME.2020.3006158.
27. Jiang, X.; Napier, C.; Hannigan, B.; Eng, J.J.; Menon, C. Estimating Vertical Ground Reaction Force during Walking Using a Single Inertial Sensor. *Sensors (Basel)* **2020**, *20*, doi:10.3390/s20154345.
28. Hendry, D.; Leadbetter, R.; McKee, K.; Hopper, L.; Wild, C.; O'Sullivan, P.; Straker, L.; Campbell, A. An Exploration of Machine-Learning Estimation of Ground Reaction Force from Wearable Sensor Data. *Sensors (Basel)* **2020**, *20*, doi:10.3390/s20030740.
29. Gurchiek, R.D.; McGinnis, R.S.; Needle, A.R.; McBride, J.M.; van Werkhoven, H. The use of a single inertial sensor to estimate 3-dimensional ground reaction force during accelerative running tasks. *J Biomech* **2017**, *61*, 263-268, doi:10.1016/j.jbiomech.2017.07.035.
30. Guo, Y.; Storm, F.; Zhao, Y.; Billings, S.A.; Pavic, A.; Mazza, C.; Guo, L.Z. A New Proxy Measurement Algorithm with Application to the Estimation of Vertical Ground Reaction Forces Using Wearable Sensors. *Sensors (Basel)* **2017**, *17*, doi:10.3390/s17102181.
31. Fukutoku, K.; Nozaki, T.; Murakami, T. Measurement of Joint Moments using Wearable Sensors. *IEEJ Journal of Industry Applications* **2020**, *9*, 125-131, doi:10.1541/ieejjia.9.125.
32. Dorschky, E.; Nitschke, M.; Seifer, A.-K.; van den Bogert, A.J.; Eskofier, B.M. Estimation of gait kinematics and kinetics from inertial sensor data using optimal control of musculoskeletal models. *Journal of biomechanics* **2019**, *95*, 109278, doi:10.1016/j.jbiomech.2019.07.022.
33. Diraneyya, M.M.; Ryu, J.; Abdel-Rahman, E.; Haas, C.T. Inertial Motion Capture-Based Whole-Body Inverse Dynamics. *Sensors (Basel)* **2021**, *21*, doi:10.3390/s21217353.
34. Dorschky, E.; Nitschke, M.; Martindale, C.F.; van den Bogert, A.J.; Koelewijn, A.D.; Eskofier, B.M. CNN-Based Estimation of Sagittal Plane Walking and Running Biomechanics From Measured and Simulated Inertial Sensor Data. *Front Bioeng Biotechnol* **2020**, *8*, 604, doi:10.3389/fbioe.2020.00604.
35. Davidson, P.; Virekunnas, H.; Sharma, D.; Piche, R.; Cronin, N. Continuous Analysis of Running Mechanics by Means of an Integrated INS/GPS Device. *Sensors (Basel)* **2019**, *19*, doi:10.3390/s19061480.
36. d'Andrea, F.; Heller, B.; James, D.; Koerger, H.; Dunn, M. Ground reaction force estimation in football using inertial measurement units during alternate lateral bounding. *Footwear Science* **2019**, *11*, S77-S78, doi:10.1080/19424280.2019.1606087.
37. Chien, K.Y.; Chang, W.G.; Chen, W.C.; Liou, R.J. Accelerometer-based prediction of ground reaction force in head-out water exercise with different exercise intensity countermovement jump. *BMC Sports Sci Med Rehabil* **2022**, *14*, 1, doi:10.1186/s13102-021-00389-8.
38. Cerfoglio, S.; Galli, M.; Tarabini, M.; Bertozzi, F.; Sforza, C.; Zago, M. Machine Learning-Based Estimation of Ground Reaction Forces and Knee Joint Kinetics from Inertial Sensors While Performing a Vertical Drop Jump. *Sensors (Basel)* **2021**, *21*, doi:10.3390/s21227709.
39. Brownjohn, J.M.W.; Chen, J.; Bocian, M.; Racic, V.; Shahabpoor, E. Using inertial measurement units to identify medio-lateral ground reaction forces due to walking and swaying. *Journal of Sound and Vibration* **2018**, *426*, 90-110, doi:10.1016/j.jsv.2018.04.019.
40. Bonnet, V.; Mazzà, C.; Fraisse, P.; Cappozzo, A. An optimization algorithm for joint mechanics estimate using inertial measurement unit data during a squat task. In Proceedings of the 2011 Annual International Conference of the IEEE Engineering in Medicine and Biology Society, Boston, MA, USA, 2011; pp. 3488-3491.
41. Hossain, M.S.B.; Guo, Z.; Choi, H. Estimation of Lower Extremity Joint Moments and 3D Ground Reaction Forces Using IMU Sensors in Multiple Walking Conditions: A Deep Learning Approach. *IEEE J Biomed Health Inform* **2023**, *27*, 2829-2840, doi:10.1109/JBHI.2023.3262164.
42. Donahue, S.R.; Hahn, M.E. Estimation of ground reaction force waveforms during fixed pace running outside the laboratory. *Front Sports Act Living* **2023**, *5*, 974186, doi:10.3389/fspor.2023.974186.

43. Bach, M.M.; Dominici, N.; Daffertshofer, A. Predicting vertical ground reaction forces from 3D accelerometry using reservoir computers leads to accurate gait event detection. *Front Sports Act Living* **2022**, *4*, 1037438, doi:10.3389/fspor.2022.1037438.
44. Havashinezhadian, S.; Chiasson-Poirier, L.; Sylvestre, J.; Turcot, K. Inertial Sensor Location for Ground Reaction Force and Gait Event Detection Using Reservoir Computing in Gait. *Int J Environ Res Public Health* **2023**, *20*, doi:10.3390/ijerph20043120.
45. Kerns, J.A.; Zwart, A.S.; Perez, P.S.; Gurchiek, R.D.; McBride, J.M. Effect of IMU location on estimation of vertical ground reaction force during jumping. *Front Bioeng Biotechnol* **2023**, *11*, 1112866, doi:10.3389/fbioe.2023.1112866.
46. Neugebauer, J.M.; Lafiandra, M. Predicting Ground Reaction Force from a Hip-Borne Accelerometer during Load Carriage. *Med Sci Sports Exerc* **2018**, *50*, 2369-2374, doi:10.1249/MSS.0000000000001686.
47. Patoz, A.; Lussiana, T.; Breine, B.; Gindre, C.; Malatesta, D. A Single Sacral-Mounted Inertial Measurement Unit to Estimate Peak Vertical Ground Reaction Force, Contact Time, and Flight Time in Running. *Sensors (Basel)* **2022**, *22*, doi:10.3390/s22030784.
48. Neugebauer, J.M.; Hawkins, D.A.; Beckett, L. Estimating youth locomotion ground reaction forces using an accelerometer-based activity monitor. *PLoS One* **2012**, *7*, e48182, doi:10.1371/journal.pone.0048182.
49. Wundersitz, D.W.; Netto, K.J.; Aisbett, B.; Gastin, P.B. Validity of an upper-body-mounted accelerometer to measure peak vertical and resultant force during running and change-of-direction tasks. *Sports Biomech* **2013**, *12*, 403-412, doi:10.1080/14763141.2013.811284.
50. Veras, L.; Diniz-Sousa, F.; Boppre, G.; Devezas, V.; Santos-Sousa, H.; Preto, J.; Vilas-Boas, J.P.; Machado, L.; Oliveira, J.; Fonseca, H. Accelerometer-based prediction of skeletal mechanical loading during walking in normal weight to severely obese subjects. *Osteoporos Int* **2020**, *31*, 1239-1250, doi:10.1007/s00198-020-05295-2.
51. Madansingh, S.I.; Murphree, D.H.; Kaufman, K.R.; Fortune, E. Assessment of gait kinetics in post-menopausal women using tri-axial ankle accelerometers during barefoot walking. *Gait Posture* **2019**, *69*, 85-90, doi:10.1016/j.gaitpost.2019.01.021.
52. LeBlanc, B.; Hernandez, E.M.; McGinnis, R.S.; Gurchiek, R.D. Continuous estimation of ground reaction force during long distance running within a fatigue monitoring framework: A Kalman filter-based model-data fusion approach. *J Biomech* **2021**, *115*, 110130, doi:10.1016/j.jbiomech.2020.110130.
53. Chaaban, C.R.; Berry, N.T.; Armitano-Lago, C.; Kiefer, A.W.; Mazzoleni, M.J.; Padua, D.A. Combining Inertial Sensors and Machine Learning to Predict vGRF and Knee Biomechanics during a Double Limb Jump Landing Task. *Sensors (Basel)* **2021**, *21*, doi:10.3390/s21134383.
54. Alcantara, R.S.; Day, E.M.; Hahn, M.E.; Grabowski, A.M. Sacral acceleration can predict whole-body kinetics and stride kinematics across running speeds. *PeerJ* **2021**, *9*, e11199, doi:10.7717/peerj.11199.
55. Veras, L.; Diniz-Sousa, F.; Boppre, G.; Moutinho-Ribeiro, E.; Resende-Coelho, A.; Devezas, V.; Santos-Sousa, H.; Preto, J.; Vilas-Boas, J.P.; Machado, L.; et al. Mechanical loading prediction through accelerometry data during walking and running. *Eur J Sport Sci* **2022**, 1-18, doi:10.1080/17461391.2022.2102437.
56. Veras, L.; Diniz-Sousa, F.; Boppre, G.; Devezas, V.; Santos-Sousa, H.; Preto, J.; Vilas-Boas, J.P.; Machado, L.; Oliveira, J.; Fonseca, H. Using Raw Accelerometer Data to Predict High-Impact Mechanical Loading. *Sensors (Basel)* **2023**, *23*, doi:10.3390/s23042246.
57. Patoz, A.; Lussiana, T.; Breine, B.; Gindre, C.; Malatesta, D. Comparison of different machine learning models to enhance sacral acceleration-based estimations of running stride temporal variables and peak vertical ground reaction force. *Sports Biomech* **2023**, 1-17, doi:10.1080/14763141.2022.2159870.
58. Liu, K.; Yan, J.; Liu, Y.; Ye, M. Noninvasive Estimation of Joint Moments with Inertial Sensor System for Analysis of STS Rehabilitation Training. *J Healthc Eng* **2018**, *2018*, 6570617, doi:10.1155/2018/6570617.
59. Mundt, M.; Thomsen, W.; Witter, T.; Koeppe, A.; David, S.; Bamer, F.; Potthast, W.; Markert, B. Prediction of lower limb joint angles and moments during gait using artificial neural networks. *Med Biol Eng Comput* **2020**, *58*, 211-225, doi:10.1007/s11517-019-02061-3.
60. Molinaro, D.D.; Kang, I.; Camargo, J.; Gombolay, M.C.; Young, A.J. Subject-Independent, Biological Hip Moment Estimation During Multimodal Overground Ambulation Using Deep Learning. *IEEE Transactions on Medical Robotics and Bionics* **2022**, *4*, 219-229, doi:10.1109/tmrb.2022.3144025.
61. van den Bogert, A.J.; Read, L.; Nigg, B.M. A method for inverse dynamic analysis using accelerometry. *Journal of biomechanics* **1996**, *29*, 949-954, doi:10.1016/0021-9290(95)00155-7.
62. Mundt, M.; Koeppe, A.; David, S.; Witter, T.; Bamer, F.; Potthast, W.; Markert, B. Estimation of Gait Mechanics Based on Simulated and Measured IMU Data Using an Artificial Neural Network. *Front Bioeng Biotechnol* **2020**, *8*, 41, doi:10.3389/fbioe.2020.00041.
63. Konrath, J.M.; Karatsidis, A.; Schepers, H.M.; Bellusci, G.; de Zee, M.; Andersen, M.S. Estimation of the Knee Adduction Moment and Joint Contact Force during Daily Living Activities Using Inertial Motion Capture. *Sensors (Basel)* **2019**, *19*, doi:10.3390/s19071681.
64. Yang, E.C.-Y.; Mao, M.-H. 3D analysis system for estimating intersegmental forces and moments exerted on human lower limbs during walking motion. *Measurement* **2015**, *73*, 171-179, doi:10.1016/j.measurement.2015.05.020.

65. Yang, E.C.-Y.; Mao, M.-H. Analytical model for estimating intersegmental forces exerted on human lower limbs during walking motion. *Measurement* **2014**, *56*, 30-36, doi:10.1016/j.measurement.2014.06.021.
66. Stetter, B.J.; Ringhof, S.; Krafft, F.C.; Sell, S.; Stein, T. Estimation of Knee Joint Forces in Sport Movements Using Wearable Sensors and Machine Learning. *Sensors (Basel)* **2019**, *19*, doi:10.3390/s19173690.
67. Bonnet, V.; Mazza, C.; Fraisse, P.; Cappozzo, A. A least-squares identification algorithm for estimating squat exercise mechanics using a single inertial measurement unit. *J Biomech* **2012**, *45*, 1472-1477, doi:10.1016/j.jbiomech.2012.02.014.
