# Supplementary material for: Estimation of Kinetics Using IMUs to Monitor and Aid in Clinical Decision-Making during ACL Rehabilitation: A Systematic Review
Source: Sensors (Basel). 2024 Mar 28;24(7):2163. doi: 10.3390/s24072163 (PMC11014074; doi:10.3390/s24072163)
Supplement: Supplementary file 1 [file sensors-24-02163-s001.zip › Supplementary_PRISMA Checklist and Quality Assessment ( Table S1, S2).pdf]

Table S1: PRISMA Checklist

Section and Topic	Item #	Checklist item	Location where item is reported
**TITLE**			
Title	1	Identify the report as a systematic review.	Page 1
**ABSTRACT**			
Abstract	2	See the PRISMA 2020 for Abstracts checklist.	-
**INTRODUCTION**			
Rationale	3	Describe the rationale for the review in the context of existing knowledge.	Page 2
Objectives	4	Provide an explicit statement of the objective(s) or question(s) the review addresses.	Page 3
**METHODS**			
Eligibility criteria	5	Specify the inclusion and exclusion criteria for the review and how studies were grouped for the syntheses.	Page 3
Information sources	6	Specify all databases, registers, websites, organisations, reference lists and other sources searched or consulted to identify studies. Specify the date when each source was last searched or consulted.	Page 3
Search strategy	7	Present the full search strategies for all databases, registers and websites, including any filters and limits used.	Page 18-19
Selection process	8	Specify the methods used to decide whether a study met the inclusion criteria of the review, including how many reviewers screened each record and each report retrieved, whether they worked independently, and if applicable, details of automation tools used in the process.	Page 3-4
Data collection process	9	Specify the methods used to collect data from reports, including how many reviewers collected data from each report, whether they worked independently, any processes for obtaining or confirming data from study investigators, and if applicable, details of automation tools used in the process.	Page 5
Data items	10a	List and define all outcomes for which data were sought. Specify whether all results that were compatible with each outcome domain in each study were sought (e.g. for all measures, time points, analyses), and if not, the methods used to decide which results to collect.	Page 5
	10b	List and define all other variables for which data were sought (e.g. participant and intervention characteristics, funding sources). Describe any assumptions made about any missing or unclear information.	Page 5
Study risk of bias assessment	11	Specify the methods used to assess risk of bias in the included studies, including details of the tool(s) used, how many reviewers assessed each study and whether they worked independently, and if applicable, details of automation tools used in the process.	Page 4-5
Effect measures	12	Specify for each outcome the effect measure(s) (e.g. risk ratio, mean difference) used in the synthesis or presentation of results.	NA
Synthesis methods	13a	Describe the processes used to decide which studies were eligible for each synthesis (e.g. tabulating the study intervention characteristics and comparing against the planned groups for each synthesis (item #5)).	NA
	13b	Describe any methods required to prepare the data for presentation or synthesis, such as handling of missing summary statistics, or data conversions.	Page 5-13
	13c	Describe any methods used to tabulate or visually display results of individual studies and syntheses.	Page 5-13
	13d	Describe any methods used to synthesize results and provide a rationale for the choice(s). If meta-analysis was performed, describe the model(s), method(s) to identify the presence and extent of statistical heterogeneity, and software package(s) used.	NA
	13e	Describe any methods used to explore possible causes of heterogeneity among study results (e.g. subgroup analysis, meta-regression).	Page 5-13
	13f	Describe any sensitivity analyses conducted to assess robustness of the synthesized results.	NA
Reporting bias assessment	14	Describe any methods used to assess risk of bias due to missing results in a synthesis (arising from reporting biases).	NA
Certainty assessment	15	Describe any methods used to assess certainty (or confidence) in the body of evidence for an outcome.	NA
**RESULTS**			
Study selection	16a	Describe the results of the search and selection process, from the number of records identified in the search to the number of studies included in the review, ideally using a flow diagram.	Page 4
	16b	Cite studies that might appear to meet the inclusion criteria, but which were excluded, and explain why they were excluded.	-

Section and Topic	Item #	Checklist item	Location where item is reported
Study characteristics	17	Cite each included study and present its characteristics.	Page 5-16
Risk of bias in studies	18	Present assessments of risk of bias for each included study.	Page 5 and Supplementary Table S2
Results of individual studies	19	For all outcomes, present, for each study: (a) summary statistics for each group (where appropriate) and (b) an effect estimate and its precision (e.g. confidence/credible interval), ideally using structured tables or plots.	Page 5-13
Results of syntheses	20a	For each synthesis, briefly summarise the characteristics and risk of bias among contributing studies.	Page 5-13
	20b	Present results of all statistical syntheses conducted. If meta-analysis was done, present for each the summary estimate and its precision (e.g. confidence/credible interval) and measures of statistical heterogeneity. If comparing groups, describe the direction of the effect.	Page 5-13
	20c	Present results of all investigations of possible causes of heterogeneity among study results.	Page 5-13
	20d	Present results of all sensitivity analyses conducted to assess the robustness of the synthesized results.	Supplementary Table S2
Reporting biases	21	Present assessments of risk of bias due to missing results (arising from reporting biases) for each synthesis assessed.	Supplementary Table S2
Certainty of evidence	22	Present assessments of certainty (or confidence) in the body of evidence for each outcome assessed.	Supplementary Table S2
**DISCUSSION**			
Discussion	23a	Provide a general interpretation of the results in the context of other evidence.	Page 15-16
	23b	Discuss any limitations of the evidence included in the review.	Page 16
	23c	Discuss any limitations of the review processes used.	Page 16
	23d	Discuss implications of the results for practice, policy, and future research.	Page 16-17
**OTHER INFORMATION**			
Registration and protocol	24a	Provide registration information for the review, including register name and registration number, or state that the review was not registered.	Page 3
	24b	Indicate where the review protocol can be accessed, or state that a protocol was not prepared.	Page 3
	24c	Describe and explain any amendments to information provided at registration or in the protocol.	Page 17
Support	25	Describe sources of financial or non-financial support for the review, and the role of the funders or sponsors in the review.	Page 17
Competing interests	26	Declare any competing interests of review authors.	Page 17
Availability of data, code and other materials	27	Report which of the following are publicly available and where they can be found: template data collection forms; data extracted from included studies; data used for all analyses; analytic code; any other materials used in the review.	Page 17

Table S2: Quality assessment checklist with explanation and scores of included papers.

Q.No	Questions	Explanation (if applicable)
#1	Is the specific purpose of the study clearly stated?	
#2	Is the research question of the study scientifically relevant?	
#3	Are descriptions of inclusion and/or exclusion criteria along with information about volunteers and/or patients reported?	Healthy/patients and complications are mentioned along with at least age, weight& height. Incomplete only when few parameters are mentioned
#4	Is data collection clearly described and reliable?	Clear description of sensor used, sampling rate, sensor placement, participant preparation, and other reference systems/instruments used. Incomplete when one or more is missing

**#5**	Are descriptions of validation tasks, and warmups clearly reported?	Description of tasks tested such that they can be replicated and easy to understand
**#6**	Is data processing clearly described and reliable?	Information on the filter, data used (only acc, gyro or additional sensor, reference system/s, and data processing to estimate reference outcome measures
**#7**	Are proposed algorithms clearly described and referenced?	
**#8**	Are outcomes reported topic relevant?	Do the result metrics match the aim of the paper?
**#9**	Were the algorithms validated for a variety of activities relevant to ACL and sports monitoring?	
**#10**	Does the work answer the scientific question stated in the aim?	Does the paper estimate all parameters stated in the aim and evaluated it on humans?
**#11**	Are the presentation of the results sufficient to assess the adequacy of the analysis?	Activities concerning the aim of the review
**#12**	Are the main findings of the study clearly described?	Are the outcomes relevant and comparable to the objective of the paper?
**#13**	Are appropriate statistical and comparison techniques to compare results to a reference system conducted and presented?	Are the results compared with the reference? Is data loss reported and information on missing data handling reported?
**#14**	Are the proposed algorithms validated for activities on a sufficient number of subjects?	Subjective based on included articles. 10 was chosen to be a good sample size for validation

**Ref. No**	**#1**	**#2**	**#3**	**#4**	**#5**	**#6**	**#7**	**#8**	**#9**	**#10**	**#11**	**#12**	**#13**	**#14**	**Final Score**
**[1]**	1	1	1	1	1	1	1	1	1	1	1	1	1	0.5	13.5
**[2]**	1	1	0.5	1	1	1	1	0.5	0.5	1	1	1	1	1	12.5
**[3]**	1	1	1	1	1	1	1	0.5	0.5	1	1	1	1	1	13
**[4]**	1	1	1	1	1	1	1	0.5	0.5	0.5	0.5	1	1	1	12
**[5]**	1	1	1	1	1	1	1	0.5	0.5	0.5	1	1	1	1	11.5
**[6]**	1	1	1	1	1	1	1	0.5	0.5	1	1	1	1	1	13
**[7]**	1	1	1	1	1	1	1	1	0.5	1	1	1	1	0.5	13
**[8]**	1	1	1	1	1	1	1	0.5	0.5	1	1	1	1	1	13
**[9]**	1	1	0.5	1	1	1	1	1	0.5	1	0.5	1	1	0	11.5
**[10]**	1	1	1	1	1	1	1	1	0.5	1	0.5	1	1	1	13
**[11]**	1	1	0.5	1	1	1	1	1	0.5	1	1	1	1	1	13
**[12]**	1	1	1	1	1	1	1	1	1	1	1	1	1	1	14
**[13]**	1	1	1	0.5	1	1	1	1	0.5	1	1	1	1	0.5	12.5
**[14]**	1	1	1	0.5	1	1	1	1	0.5	1	1	1	1	0.5	12.5
**[15]**	1	1	1	1	1	1	1	0.5	1	1	1	1	1	1	13.5
**[16]**	1	1	1	1	1	1	1	0.5	0.5	1	1	1	1	0.5	12.5
**[17]**	1	1	1	1	1	1	1	0.5	1	1	1	1	1	1	13.5
**[18]**	1	1	1	1	1	1	1	0.5	0.5	1	1	1	1	1	13
**[19]**	1	1	0.5	1	0.5	1	1	1	0.5	0.5	1	0.5	1	0	10.5
**[20]**	1	1	1	1	1	1	1	1	1	1	1	1	1	1	14
**[21]**	1	1	1	1	1	1	1	1	1	1	1	1	1	1	14
**[22]**	1	1	1	1	1	1	1	0.5	0.5	0.5	1	1	1	0.5	12
**[23]**	1	1	1	1	1	1	1	1	0.5	0.5	1	1	1	0.5	12.5
**[24]**	1	1	1	1	1	1	1	0.5	0.5	0.5	1	1	1	0.5	12
**[25]**	1	1	1	1	1	1	1	0.5	0.5	1	1	1	1	1	13

**[26]**	1	1	1	1	1	1	1	0.5	1	1	1	1	1	0	12.5
**[27]**	1	1	0.5	1	1	1	1	0.5	0.5	1	0.5	1	1	0.5	11.5
**[28]**	1	1	1	1	1	1	1	1	0.5	1	0.5	1	1	1	13
**[29]**	1	1	1	0.5	1	1	1	0.5	0.5	1	1	1	1	0.5	12
**[30]**	1	1	1	1	1	1	1	1	0.5	1	1	1	1	1	13.5
**[31]**	1	1	1	1	1	1	1	1	1	1	1	1	1	0.5	13.5
**[32]**	1	1	1	1	1	1	1	0.5	1	0.5	1	1	1	1	13
**[33]**	1	1	0.5	1	1	1	1	1	1	1	0.5	1	1	0.5	12.5
**[34]**	1	1	1	1	1	1	1	1	0.5	1	1	1	1	0.5	13
**[35]**	1	1	1	1	1	1	1	1	1	1	1	1	1	1	14
**[36]**	1	1	1	1	1	1	1	1	0.5	1	1	1	1	1	13.5
**[37]**	1	1	1	1	1	1	1	1	0.5	1	1	1	1	1	13.5
**[38]**	1	1	1	1	1	1	1	1	0.5	1	1	1	1	1	13.5
**[39]**	1	1	1	1	1	1	1	1	0.5	1	1	1	1	1	13.5
**[40]**	1	1	1	1	1	1	1	1	1	1	1	1	1	0.5	13.5
**[41]**	1	1	1	1	1	1	1	1	1	1	0.5	1	1	0.5	13
**[42]**	1	1	1	1	1	1	1	1	0.5	1	1	1	1	1	13.5
**[43]**	1	1	1	1	1	1	1	1	0.5	1	1	1	1	1	13.5
**[44]**	1	1	1	1	1	1	1	1	0.5	1	1	1	1	1	13.5
**[45]**	1	1	0.5	1	1	1	1	1	1	1	1	1	1	0.5	13
**[46]**	1	1	1	1	1	1	1	0.5	0.5	1	1	1	1	0.5	12.5
**[47]**	1	1	0.5	1	1	1	1	0.5	1	1	1	1	1	1	13
**[48]**	1	1	1	1	1	1	1	1	1	1	1	1	1	1	14
**[49]**	1	1	0.5	1	1	1	1	0.5	0.5	1	1	1	1	0.5	12
**[50]**	1	1	1	0.5	1	1	1	0.5	0.5	0.5	1	1	1	0	11
**[51]**	1	1	1	1	1	1	0.5	1	1	1	1	1	1	1	13.5
**[52]**	1	1	1	1	1	1	1	1	1	1	1	1	1	0.5	13.5
**[53]**	1	1	0.5	1	1	1	1	1	0.5	1	1	1	1	1	13
**[54]**	1	1	0.5	1	1	1	1	0.5	0.5	0.5	0.5	1	1	0.5	11
**[55]**	1	1	1	1	1	0.5	0.5	1	1	1	1	1	1	1	13
**[56]**	1	1	1	1	1	1	1	0.5	1	1	1	1	1	1	13.5
**[57]**	1	1	1	1	1	1	1	0.5	1	1	1	1	1	1	13.5
**[58]**	1	1	1	1	1	1	1	1	1	1	1	1	1	1	14
**[59]**	1	1	1	1	1	1	1	0.5	0.5	1	0.5	1	1	1	12.5
**[60]**	1	1	1	1	1	1	1	1	1	0.5	1	1	1	1	13.5
**[61]**	1	1	1	0.5	1	1	1	0.5	1	0.5	1	1	1	1	12.5
**[62]**	1	1	1	1	1	1	1	0.5	0.5	1	1	1	1	1	13
**[63]**	1	1	1	1	1	1	1	1	1	1	1	1	1	1	14
**[64]**	1	1	1	1	1	1	1	0.5	0.5	1	1	1	1	1	13
**[65]**	1	1	1	1	1	1	1	0.5	0.5	0.5	1	0.5	1	1	12
**[66]**	1	1	1	1	1	1	1	0.5	0.5	1	1	1	1	1	13
**[67]**	1	1	1	1	1	1	1	1	0.5	1	1	1	1	1	13.5
**[68]**	1	1	1	1	1	1	1	0.5	1	0.5	1	1	1	1	13
**[69]**	1	1	1	1	1	1	1	0.5	1	1	1	1	1	0.5	13
**[70]**	1	1	1	1	1	1	1	0.5	0.5	1	1	1	1	1	13
**[71]**	1	1	1	1	1	1	1	0.5	0.5	0.5	1	1	1	1	12.5

References:

1. Refai, M.I.M.; van Beijnum, B.F.; Buurke, J.H.; Veltink, P.H. Portable Gait Lab: Estimating 3D GRF Using a Pelvis IMU in a Foot IMU Defined Frame. *IEEE Trans Neural Syst Rehabil Eng* **2020**, *28*, 1308-1316, doi:10.1109/TNSRE.2020.2984809.
2. Neugebauer, J.M.; Lafiandra, M. Predicting Ground Reaction Force from a Hip-Borne Accelerometer during Load Carriage. *Med Sci Sports Exerc* **2018**, *50*, 2369-2374, doi:10.1249/MSS.0000000000001686.
3. Alcantara, R.S.; Edwards, W.B.; Millet, G.Y.; Grabowski, A.M. Predicting continuous ground reaction forces from accelerometers during uphill and downhill running: a recurrent neural network solution. *PeerJ* **2022**, *10*, e12752, doi:10.7717/peerj.12752.
4. Pogson, M.; Verheul, J.; Robinson, M.A.; Vanrenterghem, J.; Lisboa, P. A neural network method to predict task- and step-specific ground reaction force magnitudes from trunk accelerations during running activities. *Med Eng Phys* **2020**, *78*, 82-89, doi:10.1016/j.medengphy.2020.02.002.
5. Nedergaard, N.J.; Verheul, J.; Drust, B.; Etchells, T.; Lisboa, P.; Robinson, M.A.; Vanrenterghem, J. The feasibility of predicting ground reaction forces during running from a trunk accelerometry driven mass-spring-damper model. *PeerJ* **2018**, *6*, e6105, doi:10.7717/peerj.6105.
6. Patoz, A.; Lussiana, T.; Breine, B.; Gindre, C.; Malatesta, D. A Single Sacral-Mounted Inertial Measurement Unit to Estimate Peak Vertical Ground Reaction Force, Contact Time, and Flight Time in Running. *Sensors (Basel)* **2022**, *22*, doi:10.3390/s22030784.
7. Liu, K.; Yan, J.; Liu, Y.; Ye, M. Noninvasive Estimation of Joint Moments with Inertial Sensor System for Analysis of STS Rehabilitation Training. *J Healthc Eng* **2018**, *2018*, 6570617, doi:10.1155/2018/6570617.
8. Neugebauer, J.M.; Hawkins, D.A.; Beckett, L. Estimating youth locomotion ground reaction forces using an accelerometer-based activity monitor. *PLoS One* **2012**, *7*, e48182, doi:10.1371/journal.pone.0048182.
9. Nagashima, M. Prediction of Plantar Forces During Gait Using Wearable Sensors and Deep Neural Networks. In Proceedings of the 41st Annual International Conference of the IEEE Engineering in Medicine and Biology Society (EMBC), Berlin, Germany, 2019; pp. 3629-3632.
10. Mundt, M.; Johnson, W.R.; Potthast, W.; Markert, B.; Mian, A.; Alderson, J. A Comparison of Three Neural Network Approaches for Estimating Joint Angles and Moments from Inertial Measurement Units. *Sensors (Basel)* **2021**, *21*, doi:10.3390/s21134535.
11. Mundt, M.; Thomsen, W.; Witter, T.; Koeppe, A.; David, S.; Bamer, F.; Potthast, W.; Markert, B. Prediction of lower limb joint angles and moments during gait using artificial neural networks. *Med Biol Eng Comput* **2020**, *58*, 211-225, doi:10.1007/s11517-019-02061-3.
12. Molinaro, D.D.; Kang, I.; Camargo, J.; Gombolay, M.C.; Young, A.J. Subject-Independent, Biological Hip Moment Estimation During Multimodal Overground Ambulation Using Deep Learning. *IEEE Transactions on Medical Robotics and Bionics* **2022**, *4*, 219-229, doi:10.1109/tmrb.2022.3144025.
13. Yang, E.C.-Y.; Mao, M.-H. 3D analysis system for estimating intersegmental forces and moments exerted on human lower limbs during walking motion. *Measurement* **2015**, *73*, 171-179, doi:10.1016/j.measurement.2015.05.020.
14. Yang, E.C.-Y.; Mao, M.-H. Analytical model for estimating intersegmental forces exerted on human lower limbs during walking motion. *Measurement* **2014**, *56*, 30-36, doi:10.1016/j.measurement.2014.06.021.
15. Wundersitz, D.W.; Netto, K.J.; Aisbett, B.; Gastin, P.B. Validity of an upper-body-mounted accelerometer to measure peak vertical and resultant force during running and change-of-direction tasks. *Sports Biomech* **2013**, *12*, 403-412, doi:10.1080/14763141.2013.811284.
16. Wouda, F.J.; Giuberti, M.; Bellusci, G.; Maartens, E.; Reenalda, J.; van Beijnum, B.-J.F.; Veltink, P.H. Estimation of Vertical Ground Reaction Forces and Sagittal Knee Kinematics During Running Using Three Inertial Sensors. *Frontiers in Physiology* **2018**, *9*, doi:10.3389/fphys.2018.00218.
17. Verheul, J.; Gregson, W.; Lisboa, P.; Vanrenterghem, J.; Robinson, M.A. Whole-body biomechanical load in running-based sports: The validity of estimating ground reaction forces from segmental accelerations. *J Sci Med Sport* **2019**, *22*, 716-722, doi:10.1016/j.jsams.2018.12.007.
18. Veras, L.; Diniz-Sousa, F.; Boppre, G.; Devezas, V.; Santos-Sousa, H.; Preto, J.; Vilas-Boas, J.P.; Machado, L.; Oliveira, J.; Fonseca, H. Accelerometer-based prediction of skeletal mechanical loading during walking in normal weight to severely obese subjects. *Osteoporos Int* **2020**, *31*, 1239-1250, doi:10.1007/s00198-020-05295-2.

19. van den Bogert, A.J.; Read, L.; Nigg, B.M. A method for inverse dynamic analysis using accelerometry. *Journal of biomechanics* **1996**, *29*, 949-954, doi:10.1016/0021-9290(95)00155-7.
20. Stetter, B.J.; Ringhof, S.; Krafft, F.C.; Sell, S.; Stein, T. Estimation of Knee Joint Forces in Sport Movements Using Wearable Sensors and Machine Learning. *Sensors (Basel)* **2019**, *19*, doi:10.3390/s19173690.
21. Stetter, B.J.; Krafft, F.C.; Ringhof, S.; Stein, T.; Sell, S. A Machine Learning and Wearable Sensor Based Approach to Estimate External Knee Flexion and Adduction Moments During Various Locomotion Tasks. *Front Bioeng Biotechnol* **2020**, *8*, 9, doi:10.3389/fbioe.2020.00009.
22. Sharma, D.; Davidson, P.; Muller, P.; Piche, R. Indirect Estimation of Vertical Ground Reaction Force from a Body-Mounted INS/GPS Using Machine Learning. *Sensors (Basel)* **2021**, *21*, doi:10.3390/s21041553.
23. Shahabpoor, E.; Pavic, A.; Brownjohn, J.M.W.; Billings, S.A.; Guo, L.Z.; Bocian, M. Real-Life Measurement of Tri-Axial Walking Ground Reaction Forces Using Optimal Network of Wearable Inertial Measurement Units. *IEEE Trans Neural Syst Rehabil Eng* **2018**, *26*, 1243-1253, doi:10.1109/TNSRE.2018.2830976.
24. Shahabpoor, E.; Pavic, A. Estimation of vertical walking ground reaction force in real-life environments using single IMU sensor. *J Biomech* **2018**, *79*, 181-190, doi:10.1016/j.jbiomech.2018.08.015.
25. Revi, D.A.; Alvarez, A.M.; Walsh, C.J.; De Rossi, S.M.M.; Awad, L.N. Indirect measurement of anterior-posterior ground reaction forces using a minimal set of wearable inertial sensors: from healthy to hemiparetic walking. *J Neuroeng Rehabil* **2020**, *17*, 82, doi:10.1186/s12984-020-00700-7.
26. Recinos, E.; Abella, J.; Riyaz, S.; Demircan, E. Real-Time Vertical Ground Reaction Force Estimation in a Unified Simulation Framework Using Inertial Measurement Unit Sensors. *Robotics* **2020**, *9*, doi:10.3390/robotics9040088.
27. Ohtaki, Y.; Sagawa, K.; Inooka, H. A Method for Gait Analysis in a Daily Living Environment by Body-Mounted Instruments. *JSME International Journal Series C* **2001**, *44*, 1125-1132, doi:10.1299/jsmec.44.1125.
28. Noamani, A.; Nazarahari, M.; Lewicke, J.; Vette, A.H.; Rouhani, H. Validity of using wearable inertial sensors for assessing the dynamics of standing balance. *Med Eng Phys* **2020**, *77*, 53-59, doi:10.1016/j.medengphy.2019.10.018.
29. Ngoh, K.J.; Gouwanda, D.; Gopalai, A.A.; Chong, Y.Z. Estimation of vertical ground reaction force during running using neural network model and uniaxial accelerometer. *J Biomech* **2018**, *76*, 269-273, doi:10.1016/j.jbiomech.2018.06.006.
30. Mundt, M.; Koeppe, A.; David, S.; Witter, T.; Bamer, F.; Potthast, W.; Markert, B. Estimation of Gait Mechanics Based on Simulated and Measured IMU Data Using an Artificial Neural Network. *Front Bioeng Biotechnol* **2020**, *8*, 41, doi:10.3389/fbioe.2020.00041.
31. Mohamed Refai, M.I.; van Beijnum, B.F.; Buurke, J.H.; Veltink, P.H. Portable Gait Lab: Estimating Over-Ground 3D Ground Reaction Forces Using Only a Pelvis IMU. *Sensors (Basel)* **2020**, *20*, doi:10.3390/s20216363.
32. Madansingh, S.I.; Murphree, D.H.; Kaufman, K.R.; Fortune, E. Assessment of gait kinetics in post-menopausal women using tri-axial ankle accelerometers during barefoot walking. *Gait Posture* **2019**, *69*, 85-90, doi:10.1016/j.gaitpost.2019.01.021.
33. Logar, G.; Munih, M. Estimation of joint forces and moments for the in-run and take-off in ski jumping based on measurements with wearable inertial sensors. *Sensors (Basel)* **2015**, *15*, 11258-11276, doi:10.3390/s150511258.
34. Lim, H.; Kim, B.; Park, S. Prediction of Lower Limb Kinetics and Kinematics during Walking by a Single IMU on the Lower Back Using Machine Learning. *Sensors (Basel)* **2019**, *20*, doi:10.3390/s20010130.
35. Li, T.; Wang, L.; Yi, J.; Li, Q.; Liu, T. Reconstructing Walking Dynamics From Two Shank-Mounted Inertial Measurement Units. *IEEE/ASME Transactions on Mechatronics* **2021**, *26*, 3040-3050, doi:10.1109/tmech.2021.3051724.
36. Leporace, G.; Batista, L.A.; Nadal, J. Prediction of 3D ground reaction forces during gait based on accelerometer data. *Research on Biomedical Engineering* **2018**, *34*, 211-216, doi:10.1590/2446-4740.06817.
37. Leporace, G.; Batista, L.A.; Metsavaht, L.; Nadal, J. Residual analysis of ground reaction forces simulation during gait using neural networks with different configurations. In Proceedings of the 2015 37th Annual International Conference of the IEEE Engineering in Medicine and Biology Society (EMBC), Milan, Italy, 2015; pp. 2812-2815.
38. Lee, M.; Park, S. Estimation of Three-Dimensional Lower Limb Kinetics Data during Walking Using Machine Learning from a Single IMU Attached to the Sacrum. *Sensors (Basel)* **2020**, *20*, doi:10.3390/s20216277.

39. LeBlanc, B.; Hernandez, E.M.; McGinnis, R.S.; Gurchiek, R.D. Continuous estimation of ground reaction force during long distance running within a fatigue monitoring framework: A Kalman filter-based model-data fusion approach. *J Biomech* **2021**, *115*, 110130, doi:10.1016/j.jbiomech.2020.110130.
40. Konrath, J.M.; Karatsidis, A.; Schepers, H.M.; Bellusci, G.; de Zee, M.; Andersen, M.S. Estimation of the Knee Adduction Moment and Joint Contact Force during Daily Living Activities Using Inertial Motion Capture. *Sensors (Basel)* **2019**, *19*, doi:10.3390/s19071681.
41. Kodama, J.; Watanabe, T. Examination of Inertial Sensor-Based Estimation Methods of Lower Limb Joint Moments and Ground Reaction Force: Results for Squat and Sit-to-Stand Movements in the Sagittal Plane. *Sensors (Basel)* **2016**, *16*, doi:10.3390/s16081209.
42. Kim, B.; Lim, H.; Park, S. Spring-loaded inverted pendulum modeling improves neural network estimation of ground reaction forces. *J Biomech* **2020**, *113*, 110069, doi:10.1016/j.jbiomech.2020.110069.
43. Karatsidis, A.; Jung, M.; Schepers, H.M.; Bellusci, G.; de Zee, M.; Veltink, P.H.; Andersen, M.S. Musculoskeletal model-based inverse dynamic analysis under ambulatory conditions using inertial motion capture. *Medical engineering & physics* **2019**, *65*, 68-77, doi:10.1016/j.medengphy.2018.12.021.
44. Karatsidis, A.; Bellusci, G.; Schepers, H.M.; de Zee, M.; Andersen, M.S.; Veltink, P.H. Estimation of Ground Reaction Forces and Moments During Gait Using Only Inertial Motion Capture. *Sensors (Basel)* **2016**, *17*, doi:10.3390/s17010075.
45. Johnson, W.R.; Mian, A.; Robinson, M.A.; Verheul, J.; Lloyd, D.G.; Alderson, J.A. Multidimensional Ground Reaction Forces and Moments From Wearable Sensor Accelerations via Deep Learning. *IEEE Trans Biomed Eng* **2021**, *68*, 289-297, doi:10.1109/TBME.2020.3006158.
46. Jiang, X.; Napier, C.; Hannigan, B.; Eng, J.J.; Menon, C. Estimating Vertical Ground Reaction Force during Walking Using a Single Inertial Sensor. *Sensors (Basel)* **2020**, *20*, doi:10.3390/s20154345.
47. Hendry, D.; Leadbetter, R.; McKee, K.; Hopper, L.; Wild, C.; O'Sullivan, P.; Straker, L.; Campbell, A. An Exploration of Machine-Learning Estimation of Ground Reaction Force from Wearable Sensor Data. *Sensors (Basel)* **2020**, *20*, doi:10.3390/s20030740.
48. Gurchiek, R.D.; McGinnis, R.S.; Needle, A.R.; McBride, J.M.; van Werkhoven, H. The use of a single inertial sensor to estimate 3-dimensional ground reaction force during accelerative running tasks. *J Biomech* **2017**, *61*, 263-268, doi:10.1016/j.jbiomech.2017.07.035.
49. Guo, Y.; Storm, F.; Zhao, Y.; Billings, S.A.; Pavic, A.; Mazza, C.; Guo, L.Z. A New Proxy Measurement Algorithm with Application to the Estimation of Vertical Ground Reaction Forces Using Wearable Sensors. *Sensors (Basel)* **2017**, *17*, doi:10.3390/s17102181.
50. Fukutoku, K.; Nozaki, T.; Murakami, T. Measurement of Joint Moments using Wearable Sensors. *IEEJ Journal of Industry Applications* **2020**, *9*, 125-131, doi:10.1541/ieejjia.9.125.
51. Dorschky, E.; Nitschke, M.; Seifer, A.-K.; van den Bogert, A.J.; Eskofier, B.M. Estimation of gait kinematics and kinetics from inertial sensor data using optimal control of musculoskeletal models. *Journal of biomechanics* **2019**, *95*, 109278, doi:10.1016/j.jbiomech.2019.07.022.
52. Diraneyya, M.M.; Ryu, J.; Abdel-Rahman, E.; Haas, C.T. Inertial Motion Capture-Based Whole-Body Inverse Dynamics. *Sensors (Basel)* **2021**, *21*, doi:10.3390/s21217353.
53. Dorschky, E.; Nitschke, M.; Martindale, C.F.; van den Bogert, A.J.; Koelewijn, A.D.; Eskofier, B.M. CNN-Based Estimation of Sagittal Plane Walking and Running Biomechanics From Measured and Simulated Inertial Sensor Data. *Front Bioeng Biotechnol* **2020**, *8*, 604, doi:10.3389/fbioe.2020.00604.
54. Davidson, P.; Virekunnas, H.; Sharma, D.; Piche, R.; Cronin, N. Continuous Analysis of Running Mechanics by Means of an Integrated INS/GPS Device. *Sensors (Basel)* **2019**, *19*, doi:10.3390/s19061480.
55. d'Andrea, F.; Heller, B.; James, D.; Koerger, H.; Dunn, M. Ground reaction force estimation in football using inertial measurement units during alternate lateral bounding. *Footwear Science* **2019**, *11*, S77-S78, doi:10.1080/19424280.2019.1606087.
56. Chien, K.Y.; Chang, W.G.; Chen, W.C.; Liou, R.J. Accelerometer-based prediction of ground reaction force in head-out water exercise with different exercise intensity countermovement jump. *BMC Sports Sci Med Rehabil* **2022**, *14*, 1, doi:10.1186/s13102-021-00389-8.
57. Chaaban, C.R.; Berry, N.T.; Armitano-Lago, C.; Kiefer, A.W.; Mazzoleni, M.J.; Padua, D.A. Combining Inertial Sensors and Machine Learning to Predict vGRF and Knee Biomechanics during a Double Limb Jump Landing Task. *Sensors (Basel)* **2021**, *21*, doi:10.3390/s21134383.

58. Cerfoglio, S.; Galli, M.; Tarabini, M.; Bertozzi, F.; Sforza, C.; Zago, M. Machine Learning-Based Estimation of Ground Reaction Forces and Knee Joint Kinetics from Inertial Sensors While Performing a Vertical Drop Jump. *Sensors (Basel)* **2021**, *21*, doi:10.3390/s21227709.
59. Brownjohn, J.M.W.; Chen, J.; Bocian, M.; Racic, V.; Shahabpoor, E. Using inertial measurement units to identify medio-lateral ground reaction forces due to walking and swaying. *Journal of Sound and Vibration* **2018**, *426*, 90-110, doi:10.1016/j.jsv.2018.04.019.
60. Bonnet, V.; Mazza, C.; Fraisse, P.; Cappozzo, A. A least-squares identification algorithm for estimating squat exercise mechanics using a single inertial measurement unit. *J Biomech* **2012**, *45*, 1472-1477, doi:10.1016/j.jbiomech.2012.02.014.
61. Bonnet, V.; Mazzà, C.; Fraisse, P.; Cappozzo, A. An optimization algorithm for joint mechanics estimate using inertial measurement unit data during a squat task. In Proceedings of the 2011 Annual International Conference of the IEEE Engineering in Medicine and Biology Society, Boston, MA, USA, 2011; pp. 3488-3491.
62. Alcantara, R.S.; Day, E.M.; Hahn, M.E.; Grabowski, A.M. Sacral acceleration can predict whole-body kinetics and stride kinematics across running speeds. *PeerJ* **2021**, *9*, e11199, doi:10.7717/peerj.11199.
63. Hossain, M.S.B.; Guo, Z.; Choi, H. Estimation of Lower Extremity Joint Moments and 3D Ground Reaction Forces Using IMU Sensors in Multiple Walking Conditions: A Deep Learning Approach. *IEEE J Biomed Health Inform* **2023**, *27*, 2829-2840, doi:10.1109/JBHI.2023.3262164.
64. Donahue, S.R.; Hahn, M.E. Estimation of ground reaction force waveforms during fixed pace running outside the laboratory. *Front Sports Act Living* **2023**, *5*, 974186, doi:10.3389/fspor.2023.974186.
65. Bach, M.M.; Dominici, N.; Daffertshofer, A. Predicting vertical ground reaction forces from 3D accelerometry using reservoir computers leads to accurate gait event detection. *Front Sports Act Living* **2022**, *4*, 1037438, doi:10.3389/fspor.2022.1037438.
66. Veras, L.; Diniz-Sousa, F.; Boppre, G.; Moutinho-Ribeiro, E.; Resende-Coelho, A.; Devezas, V.; Santos-Sousa, H.; Preto, J.; Vilas-Boas, J.P.; Machado, L.; et al. Mechanical loading prediction through accelerometry data during walking and running. *Eur J Sport Sci* **2022**, 1-18, doi:10.1080/17461391.2022.2102437.
67. Havashinezhadian, S.; Chiasson-Poirier, L.; Sylvestre, J.; Turcot, K. Inertial Sensor Location for Ground Reaction Force and Gait Event Detection Using Reservoir Computing in Gait. *Int J Environ Res Public Health* **2023**, *20*, doi:10.3390/ijerph20043120.
68. Veras, L.; Diniz-Sousa, F.; Boppre, G.; Devezas, V.; Santos-Sousa, H.; Preto, J.; Vilas-Boas, J.P.; Machado, L.; Oliveira, J.; Fonseca, H. Using Raw Accelerometer Data to Predict High-Impact Mechanical Loading. *Sensors (Basel)* **2023**, *23*, doi:10.3390/s23042246.
69. Kerns, J.A.; Zwart, A.S.; Perez, P.S.; Gurchiek, R.D.; McBride, J.M. Effect of IMU location on estimation of vertical ground reaction force during jumping. *Front Bioeng Biotechnol* **2023**, *11*, 1112866, doi:10.3389/fbioe.2023.1112866.
70. Patoz, A.; Lussiana, T.; Breine, B.; Gindre, C.; Malatesta, D. Comparison of different machine learning models to enhance sacral acceleration-based estimations of running stride temporal variables and peak vertical ground reaction force. *Sports Biomech* **2023**, 1-17, doi:10.1080/14763141.2022.2159870.
71. Donahue, S.R.; Hahn, M.E. Estimation of gait events and kinetic waveforms with wearable sensors and machine learning when running in an unconstrained environment. *Sci Rep* **2023**, *13*, 2339, doi:10.1038/s41598-023-29314-4.
